# Preterm Birth in Caucasians Is Associated with Coagulation and Inflammation Pathway Gene Variants

**DOI:** 10.1371/journal.pone.0003283

**Published:** 2008-09-26

**Authors:** Digna R. Velez, Stephen J. Fortunato, Poul Thorsen, Salvatore J. Lombardi, Scott M. Williams, Ramkumar Menon

**Affiliations:** 1 Center for Human Genetics Research, Vanderbilt University, Nashville, Tennessee, United States of America; 2 The Perinatal Research Center, Nashville, Tennessee, United States of America; 3 Department of Obstetrics and Gynecology and Reproductive Science, Yale University, New Haven, Connecticut, United States of America; 4 Northern Atlantic Epidemiologic Alliance, University of Aarhus, Aarhus, Denmark; Baylor College of Medicine, United States of America

## Abstract

Spontaneous preterm birth (<37 weeks gestation—PTB) occurs in ∼12% of pregnancies in the United States, and is the largest contributor to neonatal morbidity and mortality. PTB is a complex disease, potentially induced by several etiologic factors from multiple pathophysiologic pathways. To dissect the genetic risk factors of PTB a large-scale high-throughput candidate gene association study was performed examining 1536 SNP in 130 candidate genes from hypothesized PTB pathways. Maternal and fetal DNA from 370 US Caucasian birth-events (172 cases and 198 controls) was examined. Single locus, haplotype, and multi-locus association analyses were performed separately on maternal and fetal data. For maternal data the strongest associations were found in genes in the complement-coagulation pathway related to decidual hemorrhage in PTB. In this pathway 3 of 6 genes examined had SNPs significantly associated with PTB. These include factor V (FV) that was previously associated with PTB, factor VII (FVII), and tissue plasminogen activator (tPA). The single strongest effect was observed in tPA marker rs879293 with a significant allelic (p = 2.30×10^−3^) and genotypic association (p = 2.0×10^−6^) with PTB. The odds ratio (OR) for this SNP was 2.80 [CI 1.77–4.44] for a recessive model. Given that 6 of 8 markers in tPA were statistically significant, sliding window haplotype analyses were performed and revealed an associating 4 marker haplotype in tPA (p = 6.00×10^−3^). The single strongest effect in fetal DNA was observed in the inflammatory pathway at rs17121510 in the interleukin-10 receptor antagonist (IL-10RA) gene for allele (p = 0.01) and genotype (p = 3.34×10^−4^). The OR for the IL-10RA genotypic additive model was 1.92 [CI 1.15–3.19] (p = 2.00×10^−3^). Finally, exploratory multi-locus analyses in the complement and coagulation pathway were performed and revealed a potentially significant interaction between a marker in FV (rs2187952) and FVII (rs3211719) (p<0.001). These results support a role for genes in both the coagulation and inflammation pathways, and potentially different maternal and fetal genetic risks for PTB.

## Introduction

Preterm birth (<37 weeks gestation—PTB) accounts for 12.0–13.0% of pregnancies in the United States [1], [2] and the rate in Caucasians has been trending upward in the last decade [3], [4]. PTB is associated with a 40-fold increase in neonatal morbidity and mortality [5]–[7]. The majority of these PTB result from spontaneous contractions (labor) of idiopathic causes [8]. It has become well established that PTB has a genetic component [9]–[14]. Family history of PTB [9], [11]–[14], twin studies that estimated heritability between 20 and 40%, [15], [16], and association between ethnicity/race and PTB [11], [17] support this conclusion. Although these data are not conclusive, they do suggest that genetic variation influences PTB susceptibility.

Data from previously published studies in both *in vivo* and *in vitro* human and animal models suggests that four primary pathogenic pathways either independently or through interactions lead to PTB [18]. The four proposed pathways are: 1) activation of maternal or fetal hypothalamic-pituitary-adrenal (HPA) axis; 2) decidual-chorioamniotic or systematic inflammation; 3) decidual hemorrhage (abruption) and 4) pathological distention of the uterus [18]. All pathways culminate in a common terminal pathway that causes the release of uterotonins, such as prostaglandins, leading to preterm labor and delivery [18].

Complex and poorly understood etiology underlying PTB and the increasing trends of PTB rates in Caucasians, led us to perform a large-scale PTB candidate gene association study on a US Caucasian population. In this case (PTB)-control (term birth after a normal pregnancy) study we examined 1536 single nucleotide polymorphisms (SNPs) in 130 candidate genes from the four hypothesized pathways. Given the complex exchange of genetic information at the maternal-fetal unit during pregnancy, both maternal and fetal DNA were analyzed. Single locus, haplotype, and multi-locus tests of association were performed.

## Methods

### Study population

Subjects were recruited at the Centennial Medical Center, Nashville, TN between September 2003 and December of 2006. Institutional Review Boards at TriStar Nashville, TN and Vanderbilt University, Nashville, TN approved this study. All included pregnancies were singleton live births. Race was identified by self-report and a questionnaire that traces ethnicity back two generations from the parents. Individuals who had more than one racial group in their ancestry were excluded from the study. Mothers between the ages of 18 and 40 were recruited. Gestational age was determined by last menstrual period and corroborated by ultrasound dating. In our study, PTB (cases) were defined as presence of regular uterine contractions (a minimum frequency of 2 contractions/10 minutes) and cervical changes followed by delivery at <36^0/7^ weeks gestation. The control group consisted of women having normal labor and delivery at term (≥37^0/7^ weeks) with no medical of obstetrical complications during pregnancy. A cut-off of <36^0/7^ weeks gestation was used in order to correct for the lack of precision of measurements of last menstrual dating and ultrasound dating. Subjects with multiple gestations, preeclampsia, preterm premature rupture of the membranes, placental previa, fetal anomalies, gestational diabetes, poly- and oligohydramnios, and other complications such as surgeries during pregnancies were excluded. DNA was collected from maternal blood and fetal cord blood.

### Demographic and clinical characteristics

Our study included 172 cases and 198 controls Caucasian birth-events including both maternal (145 cases and 194 controls) and fetal (140 cases and 179 controls) DNA from both mother-baby pairs and individual maternal and fetal samples. Demographic and clinical data were obtained from questionnaires and medical records.

In our study, microbial invasion of the amniotic cavity (MIAC) was defined either by presence of microbial 16s ribosomal DNA (TaqMan Assay, CA) detected by polymerase chain reaction (PCR) and/or clinical evidence [19], [20]. Cases with clinical evidence of MIAC were those individuals having three or more of the following criteria: abdominal tenderness, temperature >40°C, foul smelling vaginal discharge, an elevated C-reactive protein (CRP>0.8 U/ml)) or histologic chorioamnionitis.

### DNA sampling and genotyping

DNA was isolated from maternal and fetal blood samples using the Autopure automated system (Gentra Systems (Minneapolis, MN)). A total of 1536 tag single nucleotide polymorphisms (SNPs) were screened in 130 PTB candidate genes (Supplemental Table S1)(4 other genes were within 5000 kb of a candidate gene and were analyzed as members of the established candidate). We chose SNPs based on their ability to tag surrounding variants in the CEPH and Yoruba population of the HapMap database (http://www.hapmap.org), using a minor allele frequency (MAF) of 0.07 in CEPH and 0.20 in Yoruba and r^2^≥0.80, as our original study consisted of African Americans and Caucasians. Final analysis included 1432 markers after removing monomorphic markers and those markers that were not genotyped successfully. We also included functional SNPs and previously associated SNPs as selection criteria. Genotyping was performed by Illumina's GoldenGate genotyping system (http://www.illumina.com/General/pdf/LinkageIV/GOLDENGATE_ASSAY_FINAL.pdf).

### Bioinformatics tools

Marker positions (base pair - bp), marker function, and amino acid changes were identified using the SNPper (http://snpper.chip.org) database and using NCBI Build 35.1. Kyoto encyclopedia of genes and genomes (KEGG) (http://www.genome.ad.jp/kegg/pathway.html) was used to examine gene ontology and to group genes into potential biological pathways

### Statistical analysis

Clinical and demographic characteristics between cases and controls were compared, using Shapiro-Wilks tests of normality on gravidity (number of previous pregnancies), gestational age (days), gestational weight (grams - g), APGAR 1, and APGAR 5. All measurements deviated significantly from normality; as a result Mann-Whitney two-sample ranksum tests were used to compare case and control groups [21]. Standard t-tests were used to test whether maternal age differed between cases and controls. χ^2^ tests were used to test for differences in the counts of smokers and non-smokers between cases and controls. STATA 9.0 statistical software [22] was used for all analyses.

Samples analyzed represent those cleaned for Mendelian inconsistency on mother-baby pairs. Statistical tests for differences in single locus allele and genotype frequencies, for deviations from Hardy Weinberg Equilibrium (HWE) and for measurements of inbreeding coefficients (f) were calculated using Powermarker statistical software [23]. The inbreeding coefficient is a measure of deviations from expected heterozygosity under random mating and ranges from −1 to 1. Statistical significance for these analyses was determined using Fishers Exact tests. Initially, allelic and genotypic Fisher's Exact tests were performed, and only those associations with a p≤10^−3^ in either test were followed-up with additive, dominant, and recessive genotypic models, using logistic regression. For the logistic regression additive models risk was based on the presence of the minor allele with the homozygous major genotype used as the referent group. Dominant and recessive models were performed, modeling both the minor and major allele as the risk alleles. These analyses were performed using STATA 9.0 statistical software. Logistic regression analyses adjusting for confounding variables, smoking and gravidity, were then performed on the most significant associations (i.e., the best of the additive, dominant, or recessive model for those markers with p≤10^−3^). Smoking was modeled with non-smokers as the referent group and gravidity was modeled with zero previous pregnancies as the referent. Explicit tests for gene-environment interactions were not performed.

Pairwise linkage disequilibrium (LD) was characterized and haplotype frequencies were calculated using Powermarker [23], [24] and HaploView [25] statistical software. Standard summary statistics D' and r^2^ were calculated using HaploView [26]. Haplotype blocks were assigned using the D' confidence interval algorithm created by Gabriel *et al* (2002) [27]. Both Powermarker and HaploView use an EM algorithm to determine haplotype frequency distributions when phase is unknown. The Powermarker haplotype trend analysis was performed for dichotomous outcome with 2, 3 and 4 marker sliding windows, using 10,000 permutations in order to determine p - values. This analysis is a regression approach to test haplotype-trait association. The test for association then uses an F test for a specialized additive model. The strongest associated sliding window was then analyzed for haplotype specific effects. This included the calculation of Odds Ratios (OR) for each haplotype, as well as determination of PTB and term haplotype frequencies. The highest frequency haplotype was used as the baseline haplotype frequency. Only haplotypes with a frequency of 5% or more were considered for haplotype analyses and only significant haplotypes are reported.

Genes were grouped into KEGG biological process pathways and Z tests were used to determine if the total number of significant single locus allele and genotype associations within genes of a pathway deviated statistically from expected number of significant results given the number of tests and the dataset sample size. These analyses used only tag SNPs (r^2^≥0.6) in order to correct for lack of independence between markers within a gene due to LD.

Exploratory multi-locus analyses were performed, using Multifactor Dimensionality Reduction (MDR) that has been previously described in Ritchie *et al* 2001 and is available as open source software at www.epistasis.org [28].

Results are presented for the following analyses: 1) single locus tests of association with p values≤10^−3^ for either allele or genotype tests of association; 2) haplotype tests for genes with at least 1 marker with a p values≤10^−3^, 3 or more markers within the genes and with at least 1/3 of the markers statistically significant at the p = 0.05 level; 3) Z-tests within KEGG pathways testing for deviations from expected number of significant tests (either allele or genotype) given the number of tests performed.

## Results

### Baseline characteristics

Significant differences between cases and controls were observed for gestational age (days) (p<0.001), birth weight (g) (p<0.001), APGAR 1 (1 minute after birth) (p<0.001), APGAR 5 (5 minutes after birth)(p<0.001), gravidity (number of births) (p = 0.02), and smoking (p<0.001) (Table 1).

**Table 1:** Clinical and demographic information

Variable	Cases Mean (SD) or Median[IQR]	Controls Mean (SD) or Median[IQR]	P-Value 1
Gravidity (number of births)	2 [1–9]	2 [1–8]	0.02
Gestational Age (days)	239 [166–255]	274 [257–296]	<0.001
Gestational Weight(g)	2150 [370–3790]	3446 [2100–4661]	<0.001
APGAR 1	8 [1–9]	8 [4–9]	<0.001
APGAR 5	9 [1–9]	9 [7–10]	<0.001
Maternal Age(yrs)	27.33 (6.30)	28.39 (5.80)	0.10
Smoking (%)	31.77%	14.74%	<0.001

Means are reported with standard deviations reported in parentheses and medians are reported with interquartile ranges in brackets

1 P-values compare cases (PTB) to controls (term)

### Single locus tests of associations

Table 2 presents the results for the single locus allele and genotype associations and ORs for the best model at each marker. Among all of the 1432 SNPs analyzed 122 were statistically significant at the 0.05 level for either allelic or genotypic tests of associations in maternal samples and 112 were significant in fetal samples (Supplemental Table S2 and S3).

**Table 2:** Single locus association results and genotypic ORs

Population	*Gene(s)*	SNP rs#	Allele	Cases Freq.	Controls Freq.	Case v Control P		Model	OR	95% CI	Model P
						Allele	Genotype				
Maternal	*CRHBP*	rs1875999	G	0.29	0.40	3.00×10^−3^	0.02	Additive	1.37	1.08–1.72	8.00×10^−3^
		rs32897	G	0.13	0.21	4.00×10^−3^	0.02	Additive	1.68	1.14–2.49	9.00×10^−3^
		rs10055255	T	0.33	0.45	5.90×10^−4^	0.01	AAvAT&TT	1.95	1.25–3.05	3.00×10^−3^
	*FV*	rs9332624	C	0.03	0.01	3.00×10^−3^	0.01	Additive	9.79	1.19–80.48	0.03
	*IL5*	rs739718	G	0.09	0.04	4.10×10^−3^	0.01	AAvAG&GG	0.38	0.19–0.79	9.00×10^−3^
	*tPA*	rs879293*	A	0.35	0.46	2.30×10^−3^	2.00×10^−6^	GGvAG&AA	2.80	1.77–4.44	<1.00×10^−6^
	*PTGER3*	rs977214	G	0.09	0.13	0.17	4.08×10^−3^	AGvAA	0.51	0.29–0.90	0.02
		rs594454	G	0.44	0.31	1.00×10^−3^	4.00×10^−3^	Additive	1.71	1.24–2.35	1.00×10^−3^
	*SCNN1A/sTNF-R1*	rs3764874	G	0.28	0.19	3.00×10^−3^	0.01	Additive	1.70	1.19–2.44	4.00×10^−3^
Fetal	CBS	rs12329764	A	0.12	0.09	0.16	2.00×10^−3^	GGvAG&AA	0.86	0.75–0.99	0.04
	IL10RA	rs17121510	G	0.15	0.09	0.01	3.34×10^−4^	AAvAG&GG	0.43	0.25–0.74	2.00×10^−3^
	KL	rs9527025	C	0.10	0.18	4.0×10^−3^	0.02	Additive	1.91	1.15–3.19	0.01
		rs522796	C	0.50	0.38	3.00×10^−3^	2.00×10^−3^	Additive	1.52	1.17–1.96	2.00×10^−3^
	TREM1	rs6910730	G	0.15	0.07	3.00×10^−3^	3.00×10^−3^	Additive	2.30	1.34–3.95	2.00×10^−3^

1 maternal cases deviated from HWE at rs879293 (p = 0.01) andrs9772114 (p = 0.02) and fetal cases deviated at rs17121510 (p = 0.02)

2 maternal controls deviated from HWE at rs739718 (p = 0.02), rs879293 (p = 0.01), and rs977214 (p = 0.05) and fetal controls deviated at rs12329764 (p = 0.01)

* Still significant after within test Bonferroni correction

In maternal data the most significant results (p<0.001) were observed in markers from corticotrophin releasing hormone binding protein (CRHBP, OMIM # 122559), coagulation factor V (FV, OMIM # 227400), interleukin 5 (IL-5, OMIM # 600554), prostaglandin E receptor 3 (PTGER3, OMIM # 176806), and tissue plasminogen activator (tPA/PLAT, OMIM # 173370) (Tables 2 and 3). The single most significant association was seen in tPA at rs879293 (allele p = 2.30×10^−3^; genotype p = 2.00×10^−6^) with a case minor allele frequency (MAF) = 0.35 and a control MAF = 0.46. The best model for this marker was GG vs. AG & AA (Table 2) with an OR = 2.80 [CI 1.77–4.44] and p<1.00×10^−6^. Upon examining maternal genotypic associations, rs879293 in tPA remains significant after a Bonferroni correction. Six of the eight SNPs genotyped in tPA had a statistically significant single locus allelic and/or genotypic association, but the other SNPs failed to hold up to a Bonferroni correction.

**Table 3:** Maternal markers with strongest associations

*Gene Name*	*Gene Code*	Band^1^	# Markers Genotyped in Gene	dbSNP rs#	Role	Amino Acid Change	KEGG Pathway
*Corticotropin releasing hormone binding protein*	*CRHBP*	5q23.3	5	rs1875999	Exon	-	-
				rs32897	Intron	-	
				rs10055255	Intron	-	
*Coagulation factor V*	*FV*	1q24.2	26	rs9332624	Intron	-	Complement and coagulation cascade
*Interleukin 5*	*IL5*	5q23.3	5	rs739718	3′UTR	-	T cell receptor signaling pathway/, Fc epsilon RI signaling pathway, hematopoietic cell lineage, Jak-STAT signaling pathway, cytokine-cytokine receptor interaction
*Tissue plasminogen activator*	*tPA*	8p11.21	8	rs879293	Intron	-	Complement and coagulation cascade
*Prostaglandin E receptor 3, subtype EP3 isoform*	*PTGER3*	1p31.1	53	rs977214	Intron	-	Calcium signaling pathway, neuroactive ligand-receptor interaction
				rs594454	Intron	-	
*Sodium channel, nonvoltage-gated 1 alpha/Soluble tumor necrosis factor receptor 1*	*SCNN1A/sTNF-R1*	12p13.31	1	rs3764874	Intron (boundary)/Promoter	-	Taste transduction, cytokine-cytokine receptor interaction

1 NCBI build 35.1

2 Gene Ontology and KEGG pathway information obtained from SNPper (http://snpper.chip.org) and KEGG gene ontology browser

The most significant results in fetal samples were seen in markers from cystathionine-beta-synthase (CBS, OMIM # 236200), interleukin 10 receptor alpha (IL-10RA, OMIM # 146933), klotho isoform b (KL, OMIM # 604824), and triggering receptor expressed on myeloid cells 1 (TREM1, OMIM # 605085) (Table 2 and 4). The most significant association was seen in IL-10RA rs17121510 (allele p = 0.01; genotype p = 3.34×10^−4^) with the case MAF = 0.15 and a control MAF = 0.09. The most significant model for rs17121510 was AA vs. AG & GG with a protective OR = 0.43 [CI 0.25–0.74] (p = 2.00×10^−3^) (Table 2). In the fetal data the most significant ORs were for IL-10RA rs17121510, KL rs522796 (OR = 1.52 [CI 1.17–1.96]), and TREM1 rs6910730 (OR = 2.30 [CI 1.34–3.95]) all had equally significant results with p = 2.00×10^−3^; both KL rs522796 and TREM1 rs6910730 were additive models. However, none of these were significant after Bonferroni correction.

**Table 4:** Fetal markers with strongest associations

*Gene Name*	*Gene Code*	Band^1^	# Markers Genotyped in Gene	dbSNP rs#	Role	Amino Acid Change	KEGG Pathway
*Cystathionine-beta-synthase*	CBS	21q22.3	16	rs12329764	Intron (boundary)	-	Glycine, serine and threonine metabolism, methionine metabolism, Huntington's disease, selenoamino acid metabolism
*Interleukin 10 receptor, alpha precursor*	IL10RA	11q23.3	11	rs17121510	3′UTR	-	Cytokine-cytokine receptor interaction, Jak-STAT signaling pathway
*Klotho isoform b*	KL	13.q13.1	19	rs9527025	Coding exon	370 S/C	-
				rs522796	Intron	-	
*Triggering receptor expressed on myeloid cells 1*	TREM1	6p21.1	11	rs6910730	Intron	-	-

1 NCBI build 35.1

2 Gene Ontology and KEGG pathway information obtained from SNPper (http://snpper.chip.org) and KEGG gene ontology browser

Model p-values for associated markers were adjusted for demographic variables that had baseline differences between cases and controls (Table 5) such as smoking and gravidity. In maternal data all markers remained statistically significant after adjustments, with the exception of FV rs9332624, IL-5 rs739718, and PTGER3 rs977214. In fetal data all significance was lost with the exception of KL marker rs522796.

**Table 5:** Single locus association genotypic logistic regression analyses adjusted for smoking and gravidity

Population	*Gene(s)*	SNP rs#	Adjusted Model P^1^
Maternal	*CRHBP*	rs1875999	0.02
		rs32897	5.00×10^−3^
		rs10055255	6.00×10^−3^
	*FV*	rs9332624	-
	*IL-5*	rs739718	0.08
	*tPA*	rs879293	<1.00×10^−3^
	*PTGER3*	rs977214	0.14
		rs594454	3.00×10^−3^
	*SCNN1A*	rs3764874	0.03
Fetal	CBS	rs12329764	0.27
	IL-10RA	rs17121510	0.29
	KL	rs9527025	0.14
		rs522796	0.04
	TREM1	rs6910730	0.20

1 P value for model in Table 5 adjusted smoking and gravidity

Given that the presence of microbial invasion of the amniotic cavity (MIAC) in cases may influence associations, we examined markers for allele and genotype difference between cases with and without MIAC (Table 6). There were no differences for any of the markers we examined in maternal or fetal data at either the allele or genotype level except fetal marker TREM1 rs6910730 that differed between MIAC and no MIAC at both the allele (p = 0.02) and genotype level (p = 0.05).

**Table 6:** Single locus association analyses testing for allele and genotype differences between cases with and without MIAC

Population	*Gene(s)*	SNP rs#	PTB MIAC v PTB No MIAC	
			Allele P	Genotype P
Maternal	*CRHBP*	rs1875999	0.57	0.39
		rs32897	0.54	0.55
		rs10055255	0.42	0.73
	*FV*	rs9332624	0.33	0.68
	*IL-5*	rs739718	1.00	1.00
	*tPA*	rs879293	0.19	0.51
	*PTGER3*	rs977214	0.19	0.47
		rs594454	0.74	0.36
	*SCNN1A*	rs3764874	0.81	0.95
Fetal	CBS	rs12329764	0.43	0.76
	IL-10RA	rs17121510	0.87	0.86
	KL	rs9527025	1.00	0.20
		rs522796	0.17	0.06
	TREM1	rs6910730	0.02	0.05

Among the markers with p≤10^−3^, seven deviated from HWE for either cases or controls (Table 2). Five were from maternal data, one in cases at tPA rs879293 (p = 0.01) and PTGER3 rs977214 (p = 0.02) and one in control at IL-5 rs739718 (p = 0.02), tPA rs879293 (p = 0.01), and at PTGER3 rs594454 (p = 0.05). One was from fetal cases at IL-10RA marker rs17121510 (p = 0.02) and one was in fetal controls at CBS marker rs12329764 (p = 0.01). It is of note that for most situations where p<0.05 for HWE tests there is evidence that the inbreeding coefficients were in opposite directions in cases and controls. Specifically, for rs879293 in maternal samples the inbreeding coefficient, f, was 0.2313 in cases but in controls it was −0.1808, and for rs977214 f was 0.1463 in cases but −0.1420 in controls. Similarly for fetal samples, f differed in sign between cases and controls (−0.1369 and 0.2472, respectively) for rs12329764. The exception was rs739718 where the inbreeding coefficients were positive in both maternal cases and controls (0.0848 and 0.1133, respectively). Simulation studies published by Wittke-Thompson et al.[29] suggested that if inbreeding coefficients are of opposite signs in cases and controls this can be indicative of an association and not due to a genotyping error. In our data this suggests that the deviations are not likely due to genotyping error, as cases and controls were mixed on the plates for genotyping.

Details regarding the associated markers are on Table 3 for maternal and Table 4 for fetal data. Among these markers one was a coding exon only in fetal data (KL rs9527025, nonsynonymous amino acid change 370 S/C). The remaining markers were in promoters, introns, exon/intron boundary regions or synonymous changes in exons.

### Haplotype tests of association

Five genes from maternal data (CRHBP, FV, IL-5, tPA, and PTGER3) and three in fetal data (CBS, IL-10RA, and TREM1) that showed highly significant associations with PTB were analyzed for haplotype association. The full list of all markers examined for haplotype analyses are available in supplemental material (Supplemental Tables S2 and S3). Detailed LD structure for maternal and fetal genes for cases and controls are available in supplemental material (Supplemental Figure S1, S2, S3, S4, S5, S6, S7, S8, S9, S10, S11, S12, S13, S14, S15 and S16). Table 7 has the results of the haplotype association analyses with only the significant haplotypes and their ORs reported.

**Table 7:** Haplotype frequencies and OR for strongest sliding window

Population	*Gene(s)*	Markers	Global P^1^	Haplotype	Frequency		OR	95% CI	P
					Cases	Controls			
Maternal	*CRHBP*	rs32897-rs6453267-rs10055255-rs1875999	0.01	A-G-A-A	0.65	0.53	1.00	-	-
				A-G-T-G	0.10	0.18	0.45	0.27–0.74	9.00×10^−4^
	*FV*	rs12131397-rs9332624-rs9332618	<1.00×10^−3^	C-A-C	0.43	0.52	1.00	-	-
				A-A-C	0.44	0.34	1.57	1.11–2.21	8.00×10^−3^
	*IL5*	rs739719-rs739718	0.01	G-A	0.90	0.94	1.00	-	-
				T-G	0.09	0.03	2.70	1.30–5.85	4.00×10^−3^
	*tPA*	rs4471024-rs2020922-rs879293-rs2299609	6.00×10^−3^	T-T-A-C	0.32	0.42	1	-	-
				C-A-G-G	0.29	0.21	1.82	1.20–2.76	3.00×10^−3^
				T-T-G-C	0.21	0.17	1.62	1.03–2.55	0.03
	*PTGER3*	rs977214-rs6665776-rs594454	3.00×10^−3^	A-C-T	0.56	0.47	1.00	-	-
				A-C-G	0.31	0.44	0.59	0.42–0.83	2.00×10^−3^
		rs2050066-rs6424414-rs2300167	3.00×10^−3^	C-C-C	0.31	0.40	1.00	-	-
				C-T-T	0.30	0.26	1.47	0.98–2.21	0.05
				C-C-T	0.29	0.21	1.76	1.15–2.67	6.00×10^−4^
Fetal	*CBS*	rs1005584-rs6586282	9.00×10^−3^	-	-	-	-	-	-
		rs1005584-rs6586282-rs6586283	9.00×10^−3^	-	-	-	-	-	-
	*IL10RA*	rs4938467-rs11216666	0.02	T-T	0.43	0.51	1.00	-	-
				C-C	0.16	0.08	2.31	1.33–4.04	1.00×10^−3^
	*TREM1*	rs1817537-rs3804277-rs4711668	2.00×10^−3^	G-G-C	0.48	0.48	1.00	-	-
				C-A-C	0.25	0.16	1.61	1.04–2.51	0.02

1 Global P is the p-value for the haplotype sliding window

* Only haplotypes with frequencies of 5% in at least one status group and with a significant OR are presented.

* ORs are calculated comparing each haplotype to the highest frequency haplotype.

Among all haplotype sliding window analyses, maternal FV had the most significant global p value (<1.00×10^−3^) (Table 7; Supplemental Figure S7 and S8) associating intronic markers rs12131397-rs9332624-rs9332618 with PTB (cases). The A-A-C haplotype (rs12131397-rs9332624-rs9332618; OR = 1.57 [CI 1.11–2.21], p = 8.00×10^−3^) was the only significant haplotype. Two of these three markers (rs121313197 and rs9332618) are in strong LD (D' = 1) in both cases and controls (Supplemental Figure S7 and S8). The association does not appear to be main effect driven as rs9332624 is the only marker with a main effect but its MAF (0.03 in cases and 0.01 in controls) is low and does not appear to be contributing greatly to the haplotype association. tPA (p = 6.00×10^−3^) and PTGER3 (p = 3.00×10^−3^) both had very significant global p values for haplotype sliding windows. The haplotype associations observed in tPA at rs4471024-rs2020922-rs879293-rs2299609 appear to be main effect driven by rs879293 as the rs879293 G allele was common to both associated haplotypes (C-A-G-G, OR = 1.82 [CI 1.20–2.76], p = 3.00×10^−3^; T-T-G-C, OR = 1.62 [CI 1.03–2.55], p = 0.03) and the baseline haplotype included A. Two non-overlapping haplotypes were associated in PTGER3, rs977214-rs6665776-rs594454 and rs2050066-6424414-rs2300167 both located in intronic regions. The pattern of haplotype associations suggests that both associations are main effect driven; the first haplotype is driven by the effect of rs594454 and the second haplotype by rs2300167, which are in strong LD.

Examining the three genes in fetal data (Table 7; Supplemental Figure S11, S12, S13, S14, S15 and S16), the associated haplotypes were in CBS (rs1005584-rs6586282 global p = 9.00×10^−3^), CBS (rs1005584-rs6586282-rsrs6586283 global p = 9.00×10^−3^), IL-10RA (rs4938467-rs11216666 global p = 0.02), and TREM1 (rs1817537-rs3804277-rs4711668 global p = 2.00×10^−3^). In CBS only the global haplotype association was significant. IL-10RA had an associated haplotype C-C (rs4938467-rs11216666, OR = 2.31 [CI 1.33–4.04], p = 1.00×10^−3^), and this haplotype did not appear to be main effect driven. The TREM1 associated haplotype was for C-A-C (rs1817537-rs3804277-rs4711668, OR = 1.61 [CI 1.04–2.51], p = 0.02). The TREM1, C-A-C (rs1817537-rs3804277-rs4711668), haplotype association appears to be driven by the effects of haplotype C-A (rs1817537-rs3804277), as the baseline was G-G-C and rs4711668 C was common to both the associated haplotype and the baseline, suggesting that this marker is not greatly contributing to the haplotype effect.

### Evidence for pathway involvement

In the maternal data the genes in complement and coagulation pathway had a significant excess of associating SNPs in both the allele and genotype tests compared to expected results (Table 8). Several other KEGG defined pathways also had an excess of maternal associations, but only the cytokine-cytokine receptor showed an excess of associations with the fetal data.

**Table 8:** Significant results by KEGG pathway using only tags

KEGG Pathway (tags with r2≥0.60)	# Genes	Caucasian	
		Maternal	Fetal
Apoptosis	12		
Arachidonic acid metabolism	5		
Complement and coagulation cascade	6	* †	
Cytokine-cytokine receptor interactions	31	*	* †
Focal adhesion	6	*	
Hematopoietic cell lineage	12	*	
Jak-STAT signaling pathway	14		
MAPK signaling pathway	18		
Neuroactive ligand-receptor interaction	12		
T cell receptor signaling pathway	12		
Toll-like receptor signaling	14		
Type I diabetes mellitus	7		

* indicates statistically significant allelic association

† indicates a statistically significant genotypic association

### Multilocus analyses

**Figure 1:**
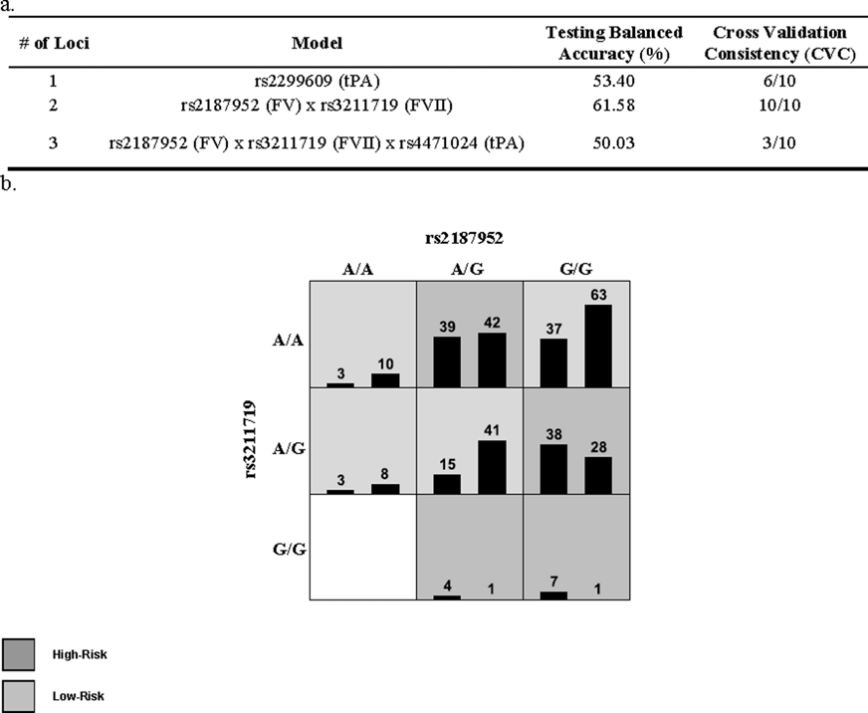
MDR complement and coagulation two locus model between FV and FVII. a. Testing accuracy and cross validation consistency for the best one to three locus models. b. Best multilocus model in the complement and coagulation pathway - Each multifactor cell is labeled as “high risk” or “low risk”. For each multifactor combination, hypothetical distributions of cases *(left bar in cell)* and controls *(right bar in cell)* are shown. Each cell represents a multilocus genotype; the genotype is labeled on the figure. The testing average balanced accuracy is 61.58% (p-value<1.00×10^−3^) with a cross-validation consistency of 10/10. Logistic regression analyses showed that the interaction for the effects of these two markers was statistically significant (p = 0.009) and the likelihood ratio test p value for including the interaction in the logistic regression model was 0.0072.

Multilocus analyses on pathways with an excess of significant associations for either allele or genotype tests (identified by Z tests analyses) revealed a statistically significant two locus model in the complement and coagulation pathway in maternal data after excluding the single most significant tPA marker (rs879293) (Figure 1). This model included markers from Factor V and Factor VII with a balanced testing accuracy of 61.58% and a cross validation consistency of 10.

## Discussion

In the present study a high-throughput candidate gene association analysis was performed on PTB candidate genes in an effort to further understand genetic factors associated with PTB in a US Caucasian sample. Comprehensive single locus and haplotype analyses were performed and revealed several interesting results in both maternal and fetal data. The most significant association in maternal data were seen in tPA, a gene involved in the complement and coagulation pathway, with other associations observed in genes involved in neuro-signaling and infection/inflammatory response. Fetal data, in contrast to maternal data, had neither highly significant associations nor an excess of significant associations in genes involved in the complement and coagulation pathways, but did have an excess of significant associations in infection/inflammatory response PTB pathways.

**Figure 2:**
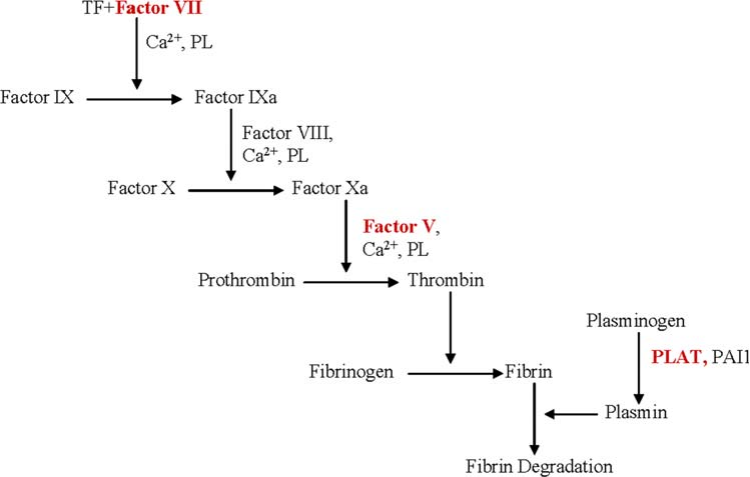
Associated genes in the complement and coagulation pathway subset. This branch of the complement and coagulation cascade had a cluster of three genes with significant results (α<0.05) at either the allele or genotype level (labeled in red). In addition to these genes we also genotyped markers in PAI1 from this branch and two genes from other branches of the complement and coagulation cascade (Soluble mannose-binding lectin 2 (MBL2), factor II (FII)); however, no markers in these genes were significant.

To further understand the significance of these variants in the pathophysiology of preterm birth a preliminary analysis was performed to putatively identify the potentially most important pathways. The complement and coagulation pathway had a significant excess of associations for both the allele and genotype tests in maternal data. The cytokine-cytokine receptor pathway was significant for both allele and genotype tests for fetal data. Upon examining the complement and coagulation pathway in more detail it was found that in the fibrinolytic branch of the pathway, three of four genes genotyped in that branch had markers that were significantly associated (Figure 2). These genes were factor VII (FVII), FV, and tPA. Two other genes upstream and downstream of this branch did not associate (soluble mannose-binding lectin (protein C) 2 (MBL2) and factor II [FII]). The only result to stand up to a Bonferroni correction was tPA, from the complement and coagulation cascade. A preliminary multi-locus analysis using multifactor dimensionality reduction (MDR) analysis revealed an interesting association between FV (rs2187952) and FVII (rs3211719) within the complement and coagulation cascade that suggests that there may be both an interaction among genes within the pathway and heterogeneity among these genes (Figure 1).

The majority of previous studies on PTB have focused on pathways contributing towards intrauterine infection [30], ignoring the potential contribution of other pathways such as decidual hemorrhage. Decidual hemorrhage is found in ∼45% of patients with PTB [31]. Expression studies of decidual tissues have observed that tissue factors and tPA have strong patterns of expression [32]–[34]. To the best of our knowledge no other studies have associated tPA markers with PTB; however, several studies have observed increased tPA and tissue factors expression in preterm compared to term human decidual tissues [34], [35]. tPA is a serine protease inhibitor in the fibrynolitic cascade (Figure 2) that converts inactive plasminogen to plasmin. The generation of plasmin is important for the degradation of components of the extra cellular matrix (ECM) by activating matrix metalloproteases (MMPs) that breaks down interstitial collagens. Although the functional relevance of tPA variants are unknown, over expression of tPA leads to plasmin production, MMP activation and ECM degradation that is commonly associated with fetal membrane rupture and cervical ripening associated with PTB. Our association with tPA seen in maternal samples supports previous findings observing elevated levels of tPA in maternally derived decidua, but not in fetally derived amnion/chorion tissues [34], [35].

Another strong association was also observed in CRHBP in maternal DNA. CRHBP plays an important role in binding of corticotrophin releasing hormone (CRH) of the HPA axis. CHRBP functions by inactivating CRH, preventing inappropriate pituitary-adrenal stimulation in pregnancy[36]. Of potential importance are the observations that tPA and the cascade of CRH have been shown to be functionally related. CRH is released by several cell types, including but not limited to neuronal cells, the gastrointestinal tracts, placenta, and fetal membranes; tPA is released in response to CRH and acts downstream of CRH receptor 1 (CRHR1), a necessary step for the activation of stress related response [37], a major etiologic factor associated with PTB. This relationship is of interest because it implicates tPA in the maternal-stress PTB pathways in addition to decidual hemorrhage pathways. If CRHBP does not function appropriately it may prevent the inactivation of CRH, causing the release of tPA and the activation of stress related response. However, despite the biological relationship between these two genes, no statistical associations were observed between them.

Single locus tests of association in fetal data were not as strong as maternal data and were not statistically significant after adjusting for the effects of confounders (gravidity and smoking) with the exception of KL (rs522796). PTB with and without MIAC did not differ with the exception of TREM1 marker rs6910730. The cytokine-cytokine receptor interaction pathway, was the only pathway showing an excess of significant findings in fetal data. The cytokine-cytokine receptor interaction pathway was also observed in maternal data, which may be the result of either maternal-fetal allele sharing or of independent associations in mother and baby; however, such relationships can only be clarified by assessment of paternal contribution by the use of a family-based study design. Although we did not have the power to detect very small effect sizes, especially after an adjustment for multiple testing, we did have the power to find associations with large effect sizes, as we did with tPA. This may be a limitation to our study. However, by finding several effects in the same pathways, using even an alpha level of 0.05 for significance, we were able to identify those pathways of the greatest importance in our data set, and reinforced the overall findings. This allowed us to look for patterns of associations, considering both large and small effects.

In conclusion, we conducted a gene-centric association study on PTB and found several interesting associations confirming several established PTB candidates. Despite basing our candidates on preterm birth pathways [18], we analyzed genes according KEGG biological pathway and found several interesting associations, including a result that stood up to a Bonferroni correction. Sample size is a major limitation to our study; however, our samples were well phenotyped and phenotypically quite homogenous. Given the patterns of associations observed within genes and by pathway, it is clear that several of the findings are consistent with the established literature. We replicated association with FV, a well established PTB candidate also found using a large-scale high throughput genotyping platform and identified new candidates [38]. This association was previously observed in a black population; however, our study suggests that it was not necessarily population specific given that we found the association in Caucasians. We stress that although our results are very promising independent replication is necessary in order to validate these associations. Our strongest association was in the decidual hemorrhage pathway, indicating that this pathway may be very important for the initiation of PTB and should be focused on in future studies.

## Supporting Information

Table S1 (0.60 MB DOC)

Table S2 (0.11 MB DOC)

Table S3 (0.10 MB DOC)

Figure S1 Maternal CRHBP Cases
(0.03 MB DOC)

Figure S2 Maternal CRHBP Controls
(0.03 MB DOC)

Figure S3 Maternal IL-5 Cases
(0.03 MB DOC)

Figure S4 Maternal IL-5 Controls
(0.03 MB DOC)

Figure S5 Maternal tPA Cases
(0.03 MB DOC)

Figure S6 Maternal tPA controls
(0.03 MB DOC)

Figure S7 Maternal FV Cases
(0.08 MB DOC)

Figure S8 Maternal FV Controls
(0.08 MB DOC)

Figure S9 Maternal PTGER3 Cases
(0.24 MB DOC)

Figure S10 Maternal PTGER3 Controls
(0.24 MB DOC)

Figure S11 Fetal CBS Cases
(0.05 MB DOC)

Figure S12 Fetal CBS Controls
(0.05 MB DOC)

Figure S13 Fetal IL-10RA Cases
(0.04 MB DOC)

Figure S14 Fetal IL-10RA Controls
(0.04 MB DOC)

Figure S15 Fetal TREM1 Cases
(0.04 MB DOC)

Figure S16 Fetal TREM1 Controls
(0.04 MB DOC)
