# Supplementary material for: Preterm Birth in Caucasians Is Associated with Coagulation and Inflammation Pathway Gene Variants
Source: PLoS One. 2008 Sep 26;3(9):e3283. doi: 10.1371/journal.pone.0003283 (PMC2553267; doi:10.1371/journal.pone.0003283)
Supplement: Table S1 — (0.60 MB DOC) [file pone.0003283.s001.doc]

Supplementary Table S1. List of all genes rs numbers for markers successfully genotyped.

| **Gene Code** | **SNP rs#** | **Gene Code** | **SNP rs#** |  | **Gene Code** | **SNP rs#** | **Gene Code** | **SNP rs#** |
| --- | --- | --- | --- | --- | --- | --- | --- | --- |
| ACE | rs7214530 | FAS | rs7901656 |  | IL6 | rs1554606 | PTGER3 | rs6685546 |
| ACE | rs4295 | FAS | rs2031611 |  | IL6 | rs11766273 | PTGER3 | rs17131465 |
| ACE | rs4305 | FAS | rs9658761 |  | IL-6 | rs1800796 | PTGER3 | rs12119442 |
| ACE | rs4311 | FAS | rs982764 |  | IL6R | rs952146 | PTGER3 | rs5702 |
| ACE | rs4362 | FAS | rs2234978 |  | IL6R | rs1552481 | PTGER3 | rs1409985 |
| ACE | rs4461142 | FAS | rs1051070 |  | IL6R | rs6427641 | PTGER3 | rs1327449 |
| ACE | rs4267385 | FAS | rs7915235 |  | IL6R | rs11265610 | PTGER3 | rs4649932 |
| ACE | rs4459610 | FASLG | rs2859242 |  | IL6R | rs1386821 | PTGER3 | rs1409981 |
| ACE | rs12451328 | FASLG | rs2639614 |  | IL6R | rs4075015 | PTGER3 | rs4147115 |
| ADH1B | rs12507573 | FASLG | rs6700734 |  | IL6R | rs4601580 | PTGER3 | rs4998697 |
| ADH1B | rs1042026 | FASLG | rs17370527 |  | IL6R | rs4845618 | PTGER3 | rs7541092 |
| ADH1B | rs17033 | FASLG | rs5030772 |  | IL6R | rs6687726 | PTGER3 | rs1409165 |
| ADH1B | rs13133908 | FASLG | rs12041613 |  | IL6R | rs7549338 | PTGER3 | rs17131487 |
| ADH1B | rs1789882 | GSTP1 | rs947895 |  | IL6R | rs4553185 | PTGER3 | rs4650094 |
| ADH1B | rs1693457 | GSTT2 | rs140199 |  | IL6R | rs4845622 | PTGER3 | rs875727 |
| ADH1B | rs4147536 | HSD11B1 | rs17389016 |  | IL6R | rs4845623 | PTGER3 | rs17541722 |
| ADH1B | rs1353621 | HSD11B1 | rs2235543 |  | IL6R | rs4537545 | PTGER3 | rs1327466 |
| ADH1B | rs1159918 | HSD11B1 | rs4844880 |  | IL6R | rs4845625 | PTGER3 | rs1887404 |
| ADH1B | rs6810842 | HSD11B1 | rs846910 |  | IL6R | rs4845374 | PTGER3 | rs17542063 |
| ADH1B | rs1229982 | HSD11B1 | rs3753519 |  | IL6R | rs11265618 | PTGER3 | rs6424410 |
| ADH1C | rs1229980 | HSD11B1 | rs846911 |  | IL6R | rs10752641 | PTGER3 | rs602383 |
| ADH1C | rs1614972 | HSD11B1 | rs11799643 |  | IL6R | rs4329505 | PTGER3 | rs578096 |
| ADH1C | rs904096 | HSD11B1 | rs12040780 |  | IL6R | rs2229238 | PTGER3 | rs6670616 |
| ADH1C | rs3762896 | HSD11B1 | rs4844488 |  | IL6R | rs4072391 | PTGER3 | rs2421735 |
| ADH1C | rs17586163 | HSD11B1 | rs846906 |  | IL6R | rs7526293 | PTGER3 | rs977214 |
| ADH1C | rs1662037 | HSD11B1 | rs6672256 |  | IL8 | rs2227538 | PTGER3 | rs6665776 |
| ADRB2 | rs1432622 | HSD11B1 | rs9430012 |  | IL8 | rs4694178 | PTGER3 | rs594454 |
| ADRB2 | rs12654778 | HSD11B1 | rs932335 |  | IL8 | rs4694637 | PTGER3 | rs2300161 |
| ADRB2 | rs1042713 | HSD17B7 | rs1780019 |  | IL8RA | rs1008563 | PTGER3 | rs5697 |
| ADRB2 | rs4705271 | HSD17B7 | rs11589262 |  | IL8RA | rs1008562 | PTGER3 | rs2072947 |
| CARD15 | rs4785224 | HSD17B7 | rs4656381 |  | IL8RA | rs2854386 | PTGER3 | rs481940 |
| CARD15 | rs2067085 | HSD17B7 | rs1039874 |  | IL8RA | rs16858856 | PTGER3 | rs3819783 |
| CARD15 | rs17312836 | HSD17B7 | rs2805053 |  | KL | rs398655 | PTGER3 | rs3819790 |
| CARD15 | rs2066843 | HSD17B7 | rs2803865 |  | KL | rs495392 | PTGER3 | rs726764 |
| CARD15 | rs2066844 | HSPA14 | rs10906772 |  | KL | rs2283368 | PTGER3 | rs1409164 |
| CARD15 | rs751271 | HSPA14 | rs11593057 |  | KL | rs526906 | PTGER3 | rs2256385 |
| CARD15 | rs2066845 | HSPA14 | rs17155992 |  | KL | rs9526983 | PTGER3 | rs2300164 |
| CARD15 | rs5743289 | HSPA14 | rs7894284 |  | KL | rs9536239 | PTGER3 | rs2050066 |
| CARD15 | rs5743291 | HSPA14 | rs9787671 |  | KL | rs577912 | PTGER3 | rs6424414 |
| CARD15 | rs7203344 | HSPA14 | rs7905174 |  | KL | rs1207362 | PTGER3 | rs2300167 |
| CARD15 | rs8056611 | HSPA14 | rs10906774 |  | KL | rs685417 | PTGER3 | rs6678886 |
| CBS | rs706208 | HSPA1A | rs1043618 |  | KL | rs1334928 | PTGER3 | rs10789314 |
| CBS | rs1051319 | HSPA1A | rs9469058 |  | KL | rs1888057 | PTGER3 | rs5693 |
| CBS | rs3788050 | HSPA1A | rs13211317 |  | KL | rs657049 | PTGER3 | rs5680 |
| CBS | rs2124459 | HSPA1B | rs2763979 |  | KL | rs9527025 | PTGER3 | rs5673 |
| CBS | rs8132811 | HSPA1B | rs2471980 |  | KL | rs2149860 | PTGER3 | rs11209736 |
| CBS | rs1005584 | HSPA1L | rs2075800 |  | KL | rs522796 | PTGER3 | rs8179390 |
| CBS | rs6586282 | HSPA1L | rs2227956 |  | KL | rs648202 | PTGER3 | rs2817864 |
| CBS | rs6586283 | HSPA4 | rs4574536 |  | KL | rs643780 | PTGER3 | rs3000466 |
| CBS | rs11203172 | HSPA4 | rs4705990 |  | KL | rs659117 | PTGES | rs11999368 |
| CBS | rs12329764 | HSPA4 | rs4616886 |  | KL | rs582524 | PTGES | rs4636306 |
| CBS | rs234705 | HSPA4 | rs7730747 |  | LST1 | rs3179004 | PTGES | rs2302821 |
| CBS | rs234709 | HSPA4 | rs10075878 |  | MBL2 | rs16933062 | PTGES | rs4837405 |
| CBS | rs2851391 | HSPA6 | rs9427401 |  | MBL2 | rs12771266 | PTGES | rs10739757 |
| CBS | rs234715 | HSPA6 | rs12129787 |  | MBL2 | rs2506 | PTGES | rs2241270 |
| CBS | rs11701048 | HSPA6 | rs4657053 |  | MBL2 | rs10082466 | PTGES | rs10988496 |
| CBS | rs1788484 | HSPA6 | rs4657054 |  | MBL2 | rs930507 | PTGFR | rs3766354 |
| CCL2 | rs1024611 | HSPA6 | rs404508 |  | MBL2 | rs1838065 | PTGFR | rs1555541 |
| CCL2 | rs1024610 | HSPA6 | rs2099684 |  | MBL2 | rs1838066 | PTGFR | rs1322934 |
| CCL2 | rs3760396 | IFNG | rs10878763 |  | MBL2 | rs10824793 | PTGFR | rs1322931 |
| CCL2 | rs4586 | IFNG | rs2069727 |  | MBL2 | rs11003123 | PTGFR | rs6424776 |
| CCL2 | rs991804 | IFNG | rs2069718 |  | MBL2 | rs10824796 | PTGFR | rs6701594 |
| CCL3 | rs1634491 | IFNG | rs2069716 |  | MBL2 | rs11003129 | PTGFR | rs12725125 |
| CCL3 | rs1851503 | IFNG | rs2069705 |  | MMP1 | rs1939008 | PTGFR | rs3766345 |
| CCL3 | rs9972960 | IGF1 | rs1520220 |  | MMP1 | rs17293823 | PTGFR | rs668005 |
| CCL3 | rs1634502 | IGFBP3 | rs2471551 |  | MMP1 | rs7945189 | PTGFR | rs622346 |
| CCL8 | rs885691 | IL10 | rs3024498 |  | MMP1 | rs2071230 | PTGFR | rs3766333 |
| CCL8 | rs1233650 | IL10 | rs3024496 |  | MMP1 | rs470747 | PTGFR | rs12074883 |
| CCL8 | rs3138034 | IL10 | rs1800872 |  | MMP1 | rs7125062 | PTGS1 | rs1330344 |
| CCL8 | rs3138035 | IL10 | rs1800896 |  | MMP1 | rs5031036 | PTGS1 | rs1213266 |
| CCL8 | rs11575060 | IL10 | rs1800890 |  | MMP1 | rs996999 | PTGS1 | rs10306135 |
| CCL8 | rs3138039 | IL10RA | rs4936414 |  | MMP1 | rs470358 | PTGS1 | rs7866582 |
| CCL8 | rs4794999 | IL10RA | rs2512143 |  | MMP1 | rs514921 | PTGS1 | rs3842788 |
| CD14 | rs4914 | IL10RA | rs4252254 |  | MMP1 | rs1155764 | PTGS1 | rs10306150 |
| CD14 | rs2569190 | IL10RA | rs4252270 |  | MMP1 | rs484915 | PTGS1 | rs4273915 |
| CD14 | rs2569193 | IL10RA | rs2229113 |  | MMP2 | rs243866 | PTGS1 | rs4240474 |
| COL1A1 | rs2586482 | IL10RA | rs9610 |  | MMP2 | rs1477017 | PTGS1 | rs3842798 |
| COL1A1 | rs2586485 | IL10RA | rs2508445 |  | MMP2 | rs865094 | PTGS1 | rs10306153 |
| COL1A1 | rs1061237 | IL10RA | rs947889 |  | MMP2 | rs11646643 | PTGS1 | rs4836885 |
| COL1A1 | rs2277632 | IL10RA | rs4938467 |  | MMP2 | rs1053605 | PTGS1 | rs9299282 |
| COL1A1 | rs2586488 | IL10RA | rs11216666 |  | MMP2 | rs866770 | PTGS1 | rs12238505 |
| COL1A1 | rs2075559 | IL10RA | rs17121510 |  | MMP2 | rs2241145 | PTGS1 | rs3842801 |
| COL1A1 | rs2857396 | IL10RB | rs2284552 |  | MMP2 | rs243845 | PTGS1 | rs10306184 |
| COL1A1 | rs2696247 | IL10RB | rs962859 |  | MMP2 | rs243842 | PTGS1 | rs10306188 |
| COL1A1 | rs2269336 | IL10RB | rs2834168 |  | MMP2 | rs183112 | PTGS1 | rs10306202 |
| COL1A1 | rs1107946 | IL10RB | rs2834170 |  | MMP2 | rs1992116 | PTGS2 | rs2066826 |
| COL1A2 | rs11765563 | IL10RB | rs2243498 |  | MMP2 | rs11639960 | PTGS2 | rs2745557 |
| COL1A2 | rs388625 | IL10RB | rs2834172 |  | MMP2 | rs243836 | PTGS2 | rs689466 |
| COL1A2 | rs3814967 | IL10RB | rs765429 |  | MMP2 | rs243834 | PTGS2 | rs689462 |
| COL1A2 | rs1800222 | IL10RB | rs2276223 |  | MMP2 | rs11541998 | PTGS2 | rs12042763 |
| COL1A2 | rs411717 | IL10RB | rs735299 |  | MMP2 | rs243832 | PTGS2 | rs10911905 |
| COL1A2 | rs420257 | IL10RB | rs999261 |  | [MMP2](http://snpper.chip.org/bio/show-gene/7384) | rs243831 | PTGS2 | rs2179555 |
| COL1A2 | rs760043 | IL10RB | rs999259 |  | MMP2 | rs9922534 | PTPN22 | rs2476601 |
| COL1A2 | rs406226 | IL10RB | rs1058867 |  | MMP2 | rs2241148 | SCGB1A1 | rs10897270 |
| COL1A2 | rs3763466 | IL10RB | rs8178565 |  | MMP2 | rs8054459 | SCGB1A1 | rs3741240 |
| COL1A2 | rs17166249 | IL10RB | rs6517158 |  | MMP2 | rs2192853 | SCGB1A1 | rs17145874 |
| COL1A2 | rs389328 | IL10RB | rs2834175 |  | MMP3 | rs569444 | SCNN1A/sTNF-R1 | rs3764874 |
| COL1A2 | rs42521 | IL13 | rs3091307 |  | MMP3 | rs650108 | SERPINE1 | rs6950982 |
| COL1A2 | rs42523 | IL13 | rs1295686 |  | MMP3 | rs520540 | SERPINE1 | rs2227631 |
| COL1A2 | rs42524 | IL13 | rs848 |  | MMP3 | rs522616 | SERPINE1 | rs2227667 |
| COL1A2 | rs2621213 | IL13 | rs1295683 |  | MMP3 | rs645419 | SERPINE1 | rs2227672 |
| COL1A2 | rs2521205 | IL13 | rs2243204 |  | MMP8 | rs1276284 | SERPINE1 | rs2070682 |
| COL1A2 | rs7781954 | IL13 | rs2243209 |  | MMP8 | rs2508383 | SERPINE1 | rs1050813 |
| COL1A2 | rs3736638 | IL15 | rs12508866 |  | MMP8 | rs1939020 | SERPINE1 | rs11560324 |
| COL1A2 | rs42527 | IL15 | rs1519551 |  | MMP8 | rs17099443 | SERPINH1 | rs646474 |
| COL1A2 | rs369982 | IL15 | rs17461269 |  | MMP8 | rs1940475 | SERPINH1 | rs646851 |
| COL1A2 | rs42528 | IL15 | rs1519552 |  | MMP8 | rs11225394 | SERPINH1 | rs649257 |
| COL1A2 | rs4266 | IL15 | rs7698675 |  | MMP8 | rs6590985 | SLC23A1 | rs6596471 |
| COL1A2 | rs2472 | IL15 | rs13117878 |  | MMP8 | rs10895354 | SLC23A1 | rs6596473 |
| COL1A2 | rs42531 | IL15 | rs12498901 |  | MMP9 | rs4810482 | SLC23A1 | rs11950646 |
| COL1A2 | rs441051 | IL15 | rs6850492 |  | MMP9 | rs8113877 | SLC6A4 | rs7224199 |
| COL1A2 | rs400218 | IL15 | rs1907949 |  | MMP9 | rs6104420 | SLC6A4 | rs1042173 |
| COL1A2 | rs7804898 | IL15 | rs17007610 |  | MMP9 | rs3918260 | SLC6A4 | rs3794808 |
| COL1A2 | rs413826 | IL15 | rs6537061 |  | MTHFD1 | rs1956545 | SLC6A4 | rs140701 |
| COL1A2 | rs10046552 | IL15 | rs10833 |  | MTHFD1 | rs3783731 | SLC6A4 | rs140700 |
| COL1A2 | rs6465412 | IL18 | rs543810 |  | MTHFD1 | rs1950902 | SLC6A4 | rs2020942 |
| COL1A2 | rs12668754 | IL18 | rs5744280 |  | MTHFD1 | rs17751556 | SLC6A4 | rs6354 |
| COL1A2 | rs11764718 | IL18 | rs360722 |  | MTHFD1 | rs2295640 | SLC6A4 | rs25528 |
| COL1A2 | rs1062394 | IL18 | rs4937113 |  | MTHFD1 | rs17824591 | SLC6A4 | rs2020936 |
| COL1A2 | rs11982782 | IL18 | rs2043055 |  | MTHFD1 | rs1885031 | SLC6A4 | rs12150214 |
| COL1A2 | rs13234022 | IL18 | rs1946519 |  | MTHFD1 | rs8016556 | SLC6A4 | rs4251417 |
| COL3A1 | rs2138533 | IL18 | rs5744222 |  | MTHFD1 | rs2236225 | [SLC6A4](http://snpper.chip.org/bio/show-gene/8165) | rs9903602 |
| COL3A1 | rs11887092 | IL18 | rs1293344 |  | MTHFD1 | rs3818239 | SLC6A4 | rs16965628 |
| COL3A1 | rs7607614 | IL1A | rs17561 |  | MTHFD1 | rs11849530 | SLC6A4 | rs2020933 |
| COL3A1 | rs1878201 | IL1A | rs2856838 |  | MTHFD1 | rs1256146 | TCN2 | rs2267163 |
| COL3A1 | rs1516454 | IL1A | rs1878321 |  | MTHFD1 | rs745686 | TGFB1 | rs10417924 |
| COL3A1 | rs1914037 | IL1B | rs2853550 |  | MTHFR | rs4846048 | TGFB1 | rs8179181 |
| COL3A1 | rs17358825 | IL1B | rs1143643 |  | MTHFR | rs4846049 | TGFB1 | rs1982072 |
| COL3A1 | rs16830973 | IL1B | rs1143634 |  | MTHFR | rs1476413 | TIMP3 | rs5754289 |
| COL3A1 | rs7576108 | IL1B | rs1143630 |  | MTHFR | rs1801131 | TIMP3 | rs9606994 |
| COL3A1 | rs3736487 | IL1B | rs1143627 |  | MTHFR | rs12121543 | TIMP3 | rs9619311 |
| COL3A1 | rs3134656 | IL1B | rs1143623 |  | MTHFR | rs1994798 | TIMP3 | rs130274 |
| COL3A1 | rs12693525 | IL1B | rs4848306 |  | MTHFR | rs1801133 | TIMP3 | rs11704261 |
| COL3A1 | rs13306267 | IL1R1 | rs3917225 |  | MTHFR | rs17421462 | TIMP3 | rs738992 |
| COL3A1 | rs7579903 | IL1R1 | rs949963 |  | MTHFR | rs17421511 | TIMP3 | rs242089 |
| COL3A1 | rs2271682 | IL1R1 | rs3771202 |  | MTHFR | rs4846052 | TIMP3 | rs130287 |
| COL3A1 | rs10191859 | IL1R1 | rs2287047 |  | MTHFR | rs11121832 | TIMP3 | rs242088 |
| COL3A1 | rs10204508 | IL1R1 | rs3917254 |  | MTHFR | rs9651118 | TIMP3 | rs130290 |
| COL3A1 | rs2203602 | IL1R1 | rs3917273 |  | MTHFR | rs17367504 | TIMP3 | rs130293 |
| COL3A1 | rs3134646 | IL1R1 | rs3917292 |  | MTHFR | rs3753582 | TIMP3 | rs5749524 |
| COL3A1 | rs4667256 | IL1R1 | rs2160227 |  | MTHFR | rs3737964 | TIMP3 | rs242082 |
| COL3A1 | rs4667258 | IL1R1 | rs3917296 |  | NAT1 | rs11203942 | TIMP3 | rs80272 |
| COL5A1 | rs12002679 | IL1R1 | rs951193 |  | NAT1 | rs10888150 | TIMP3 | rs242078 |
| COL5A1 | rs4341231 | IL1R1 | rs3917304 |  | NAT1 | rs7017402 | TIMP3 | rs242076 |
| COL5A1 | rs3124291 | IL1R1 | rs3917306 |  | NAT1 | rs4298522 | TIMP3 | rs130300 |
| COL5A1 | rs3128597 | IL1R1 | rs3171845 |  | NAT1 | rs9325827 | TIMP3 | rs130301 |
| COL5A1 | rs3124311 | IL1R1 | rs2110726 |  | NAT1 | rs17126350 | TIMP3 | rs242072 |
| COL5A1 | rs4842151 | IL1R1 | rs3732131 |  | NAT1 | rs4921880 | TIMP3 | rs4504 |
| COL5A1 | rs11103509 | IL1R1 | rs3917332 |  | NAT1 | rs7003890 | TIMP3 | rs135029 |
| COL5A1 | rs4842157 | IL1R2 | rs11884283 |  | NAT1 | rs8190837 | TIMP3 | rs5754312 |
| COL5A1 | rs4842158 | IL1R2 | rs6543105 |  | NAT1 | rs8190870 | TIMP3 | rs2267183 |
| COL5A1 | rs4842161 | IL1R2 | rs12467316 |  | NAT2 | rs4646246 | TIMP3 | rs1427378 |
| COL5A1 | rs3124932 | IL1R2 | rs4141134 |  | NAT2 | rs7832071 | TIMP3 | rs1427376 |
| COL5A1 | rs12005720 | IL1R2 | rs4851520 |  | NAT2 | rs1801280 | TIMP3 | rs9862 |
| COL5A1 | rs3128621 | IL1R2 | rs4851521 |  | NAT2 | rs1799929 | TIMP3 | rs137485 |
| COL5A1 | rs4842167 | IL1R2 | rs4851522 |  | NAT2 | rs1208 | TIMP3 | rs5749527 |
| COL5A1 | rs3811161 | IL1R2 | rs4321386 |  | NAT2 | rs721398 | TIMP3 | rs137487 |
| COL5A1 | rs11999194 | IL1R2 | rs1108338 |  | NAT2 | rs721399 | TIMP3 | rs5749529 |
| COL5A1 | rs3811153 | IL1R2 | rs7561191 |  | NFKB1 | rs980455 | TIMP3 | rs137489 |
| COL5A1 | rs3811152 | IL1R2 | rs4851526 |  | NFKB1 | rs3774933 | TIMP3 | rs2040435 |
| COL5A1 | rs3811151 | IL1R2 | rs4851527 |  | NFKB1 | rs1599961 | TIMP4 | rs99365 |
| COL5A1 | rs10776908 | IL1R2 | rs2302589 |  | NFKB1 | rs1585213 | TIMP4 | rs3773364 |
| COL5A1 | rs10745387 | IL1R2 | rs3218861 |  | NFKB1 | rs230528 | TIMP4 | rs4684841 |
| COL5A1 | rs3827848 | IL1R2 | rs2160140 |  | NFKB1 | rs4648011 | TIMP4 | rs3755724 |
| COL5A1 | rs4842172 | IL1R2 | rs3218883 |  | NFKB1 | rs13117745 | TLR2 | rs1898830 |
| COL5A1 | rs4841937 | IL1R2 | rs3218927 |  | NFKB1 | rs1801 | TLR2 | rs4696483 |
| COL5A1 | rs4842174 | IL1R2 | rs2072474 |  | NFKB1 | rs4648058 | TLR2 | rs7656411 |
| COL5A1 | rs10114036 | IL1R2 | rs3218979 |  | NFKB1 | rs3755867 | TLR2 | rs1337 |
| COL5A1 | rs11103543 | IL1R2 | rs733498 |  | NFKB1 | rs4648090 | TLR3 | rs4862632 |
| COL5A1 | rs13946 | IL1R2 | rs3218987 |  | NFKB1 | rs3774968 | TLR3 | rs5743303 |
| COL5A2 | rs6760780 | IL1R2 | rs7589525 |  | NFKB1 | rs3817685 | TLR3 | rs5743305 |
| COL5A2 | rs3923384 | IL1R2 | rs4851531 |  | NFKB1 | rs4648135 | TLR3 | rs11721827 |
| COL5A2 | rs6434317 | IL1RAP | rs9817203 |  | NFKB1 | rs4648141 | TLR3 | rs5743312 |
| COL5A2 | rs6434322 | IL1RAP | rs6800625 |  | NFKB1 | rs1609798 | TLR3 | rs7668666 |
| COL5A2 | rs10165260 | IL1RAP | rs7628333 |  | NFKB1 | rs7674640 | TLR3 | rs3775292 |
| COL5A2 | rs7420331 | IL1RAP | rs3821744 |  | NFKB1 | rs997476 | TLR3 | rs3775291 |
| COL5A2 | rs13024858 | IL1RAP | rs7615368 |  | NFKB1 | rs10489113 | TLR3 | rs10025405 |
| COL5A2 | rs6752781 | IL1RAP | rs7615533 |  | NFKB2 | rs11574845 | TLR3 | rs4862633 |
| COL5A2 | rs1515864 | IL1RAP | rs3773994 |  | NFKB2 | rs7897947 | TLR4 | rs10759930 |
| COL5A2 | rs9288163 | IL1RAP | rs9290936 |  | NFKB2 | rs1056890 | TLR4 | rs16906053 |
| COL5A2 | rs10191420 | IL1RAP | rs9849030 |  | NFKBIA | rs3138056 | TLR4 | rs2770150 |
| COL5A2 | rs1399991 | IL1RAP | rs2059020 |  | NFKBIA | rs696 | TLR4 | rs10759932 |
| COL5A2 | rs10497699 | IL1RAP | rs2361832 |  | NFKBIA | rs3138045 | TLR4 | rs1927911 |
| COL5A2 | rs12611950 | IL1RAP | rs7626071 |  | NFKBIB | rs11575002 | TLR4 | rs1927907 |
| COL5A2 | rs11691604 | IL1RAP | rs3773990 |  | NFKBIB | rs2053071 | TLR4 | rs2149356 |
| [CRH](http://snpper.chip.org/bio/show-gene/18919) | rs10105164 | IL1RAP | rs2193880 |  | NFKBIB | rs2241704 | TLR4 | rs7869402 |
| CRH | rs6996265 | IL1RAP | rs3773989 |  | NFKBIB | rs2241705 | TLR4 | rs11536889 |
| CRH | rs3176921 | IL1RAP | rs1988743 |  | NFKBIB | rs3136641 | TLR4 | rs1927906 |
| CRH | rs6472257 | IL1RAP | rs4686554 |  | NFKBIB | rs9636109 | TLR4 | rs11536898 |
| CRH | rs7839698 | IL1RAP | rs16865597 |  | NFKBIB | rs11083487 | TLR4 | rs1554973 |
| CRH | rs10098823 | IL1RAP | rs2885370 |  | NFKBIB | rs3136646 | TLR4 | rs7856729 |
| CRHBP | rs32897 | IL1RAP | rs3773983 |  | NFKBIE | rs1875324 | TLR7 | rs5741880 |
| CRHBP | rs6453267 | IL1RAP | rs3773982 |  | NFKBIE | rs520639 | TLR7 | rs1731478 |
| CRHBP | rs10055255 | IL1RAP | rs3773981 |  | NFKBIE | rs2282151 | TLR7 | rs179021 |
| CRHBP | rs1875999 | IL1RAP | rs9883249 |  | NFKBIE | rs730775 | TLR7 | rs1731479 |
| CRHBP | rs10514082 | IL1RAP | rs9845825 |  | NFKBIE | rs483536 | TLR7 | rs5743740 |
| CRHR1 | rs17689966 | IL1RAP | rs2241343 |  | NOS3 | rs10277237 | TLR7 | rs179016 |
| CRHR1 | rs878887 | IL1RAP | rs9877268 |  | NOS3 | rs12703107 | TLR7 | rs179012 |
| CRHR2 | rs4722999 | IL1RAP | rs3773977 |  | NOS3 | rs1800783 | TLR7 | rs179011 |
| CRHR2 | rs12701020 | IL1RAP | rs3773976 |  | NOS3 | rs1799983 | TLR7 | rs179009 |
| CRHR2 | rs7812133 | IL1RAP | rs4687151 |  | NOS3 | rs3918227 | TLR7 | rs179008 |
| CRHR2 | rs973002 | IL1RAP | rs11929157 |  | NOS3 | rs743507 | TLR7 | rs5743780 |
| CRHR2 | rs929377 | IL1RAP | rs10937439 |  | NOS3 | rs2373929 | TLR7 | rs864058 |
| CRHR2 | rs2240404 | IL1RAP | rs10937442 |  | NR3C1 | rs17287758 | TLR7 | rs179007 |
| CRHR2 | rs2190242 | IL1RAP | rs2161058 |  | NR3C1 | rs10482682 | TLR7 | rs179006 |
| CRHR2 | rs2284217 | IL1RAP | rs1035347 |  | NR3C1 | rs4986593 | TLR7 | rs5935438 |
| CRHR2 | rs6462219 | IL1RAP | rs1559018 |  | NR3C1 | rs33388 | TLR8 | rs178996 |
| CRHR2 | rs2284219 | IL1RAP | rs12053868 |  | NR3C1 | rs17100236 | TLR8 | rs3761621 |
| CRHR2 | rs2267716 | IL1RAP | rs6444435 |  | NR3C1 | rs2918417 | TLR8 | rs3761623 |
| CRHR2 | rs2267717 | IL1RAP | rs4687154 |  | NR3C1 | rs2963155 | TLR8 | rs5741883 |
| CRP | rs3093066 | IL1RAP | rs10513854 |  | NR3C1 | rs9324918 | TLR8 | rs3764880 |
| CRP | rs1800947 | IL1RAP | rs7628250 |  | NR3C1 | rs9324921 | TLR8 | rs17256081 |
| CRP | rs1417938 | IL1RAP | rs3773958 |  | NR3C1 | rs4634384 | TLR8 | rs1548731 |
| CTLA4 | rs231770 | IL1RAP | rs3773953 |  | NR3C1 | rs9324924 | TLR8 | rs4830807 |
| CTLA4 | rs16840252 | IL1RAP | rs1469007 |  | NR3C1 | rs7701443 | TLR8 | rs5741886 |
| CTLA4 | rs11571317 | IL1RAP | rs9875362 |  | NR3C1 | rs4244032 | TLR8 | rs5744068 |
| CTLA4 | rs5742909 | IL1RAP | rs6781037 |  | NR3C1 | rs4607376 | TLR8 | rs2159377 |
| CTLA4 | rs231777 | IL1RAP | rs6765375 |  | NR3C1 | rs13182800 | TLR8 | rs2407992 |
| CTLA4 | rs231779 | IL1RAP | rs9821002 |  | NR3C1 | rs4912911 | TLR8 | rs3747414 |
| CTLA4 | rs3087243 | IL1RAP | rs759783 |  | NR3C1 | rs12656106 | TLR8 | rs5741890 |
| CTLA4 | rs231726 | IL1RAP | rs4140711 |  | NR3C1 | rs12655166 | TLR8 | rs16987224 |
| CTLA4 | rs231727 | IL1RAP | rs9290939 |  | PAFAH1B1 | rs6502385 | TLR9 | rs352143 |
| CYP19A1 | rs4775932 | IL1RAP | rs1015704 |  | PAFAH1B1 | rs7209407 | TNF | rs2844482 |
| CYP19A1 | rs4275794 | IL1RAP | rs1015705 |  | PAFAH1B1 | rs7213463 | TNF | rs1800683 |
| CYP19A1 | rs2899470 | IL1RAP | rs4687163 |  | PAFAH1B1 | rs3785958 | TNF | rs2229094 |
| CYP19A1 | rs16964201 | IL1RAP | rs929729 |  | PAFAH1B1 | rs1029744 | TNF | rs1799964 |
| CYP19A1 | rs2899472 | IL1RAP | rs1024941 |  | PAFAH1B1 | rs11078302 | TNF | rs1800629 |
| CYP19A1 | rs12439137 | IL1RAP | rs1024946 |  | PAFAH1B1 | rs12938775 | TNF | rs769178 |
| CYP19A1 | rs4775934 | IL1RAP | rs1024949 |  | PAFAH1B1 | rs2317297 | TNFRSF1A | rs740841 |
| CYP19A1 | rs2899473 | IL1RAP | rs7626795 |  | PAFAH1B1 | rs7223411 | TNFRSF1A | rs2302350 |
| CYP19A1 | rs4775935 | IL1RAP | rs4624606 |  | PAFAH1B1 | rs4790353 | TNFRSF1A | rs1800693 |
| CYP19A1 | rs3784308 | IL1RAP | rs9847868 |  | PAFAH1B1 | rs4790355 | TNFRSF1A | rs1860545 |
| CYP19A1 | rs700518 | IL1RAP | rs9821122 |  | PAFAH1B1 | rs4790356 | TNFRSF1A | rs4149578 |
| CYP19A1 | rs17703883 | IL1RAP | rs4320092 |  | PAFAH1B2 | rs4938347 | TNFRSF1A | rs4149577 |
| CYP19A1 | rs726547 | IL1RAP | rs9831803 |  | PAFAH1B2 | rs3736120 | TNFRSF1A | rs4149622 |
| CYP19A1 | rs10519295 | IL1RAP | rs7650510 |  | PGEA1 | rs6001188 | TNFRSF1A | rs4149570 |
| CYP19A1 | rs727479 | IL1RAP | rs11915384 |  | PGEA1 | rs6519132 | TNFRSF1A | rs11064145 |
| CYP19A1 | rs10459592 | IL1RN | rs17042917 |  | PGEA1 | rs5757231 | TNFRSF1B | rs590368 |
| CYP19A1 | rs12591359 | IL1RN | rs315920 |  | PGEA1 | rs6001193 | TNFRSF1B | rs522807 |
| CYP19A1 | rs767199 | IL1RN | rs4251961 |  | PGR | rs11224561 | TNFRSF1B | rs652625 |
| CYP19A1 | rs12911554 | IL1RN | rs2637988 |  | PGR | rs471767 | TNFRSF1B | rs496888 |
| CYP19A1 | rs17703982 | IL1RN | rs928940 |  | PGR | rs563656 | TNFRSF1B | rs976881 |
| CYP19A1 | rs7172156 | IL1RN | rs3213448 |  | [PGR](http://snpper.chip.org/bio/show-gene/3845) | rs504372 | TNFRSF1B | rs3766730 |
| CYP19A1 | rs11856927 | IL1RN | rs1794066 |  | PGR | rs578029 | TNFRSF1B | rs616645 |
| CYP19A1 | rs4545755 | IL1RN | rs380092 |  | PGR | rs635984 | TNFRSF1B | rs816050 |
| CYP19A1 | rs1054984 | IL1RN | rs579543 |  | PGR | rs11224575 | TNFRSF1B | rs474247 |
| CYP19A1 | rs4614671 | IL1RN | rs315951 |  | PGR | rs492457 | TNFRSF1B | rs1201157 |
| CYP19A1 | rs12050767 | IL1RN | rs315949 |  | PGR | rs518382 | TNFRSF1B | rs653667 |
| CYP19A1 | rs749292 | IL1RN | rs315946 |  | PGR | rs553272 | TNFRSF1B | rs1061622 |
| CYP19A1 | rs17647719 | IL1RN | rs315943 |  | PGR | rs660149 | TNFRSF1B | rs5746051 |
| CYP19A1 | rs2305707 | IL1RN | rs315942 |  | PGR | rs653752 | TNFRSF1B | rs5746053 |
| CYP19A1 | rs1902586 | IL2 | rs10027390 |  | PGR | rs538915 | TNFRSF1B | rs235219 |
| CYP19A1 | rs16953058 | IL2 | rs2069772 |  | PGR | rs503362 | TNFRSF1B | rs1061624 |
| CYP19A1 | rs936306 | IL2 | rs2069771 |  | PGR | rs542384 | TNFRSF1B | rs1061628 |
| CYP19A1 | rs936307 | IL2 | rs2069779 |  | PGR | rs555572 | TNFRSF1B | rs1061631 |
| CYP19A1 | rs2470176 | IL2 | rs2069778 |  | PGR | rs11224589 | TNFRSF1B | rs235214 |
| CYP19A1 | rs3764221 | IL2 | rs2069762 |  | PGR | rs619487 | TRAF2 | rs10781520 |
| CYP19A1 | rs17523880 | IL2 | rs4833248 |  | PGR | rs537681 | TRAF2 | rs2784075 |
| CYP19A1 | rs2470152 | IL2RA | rs10795737 |  | PGR | rs518162 | TRAF2 | rs908831 |
| CYP19A1 | rs17523922 | IL2RA | rs12359875 |  | PGR | rs507141 | TREM1 | rs16894387 |
| CYP19A1 | rs16964258 | IL2RA | rs12722605 |  | PGRMC1 | rs2499043 | TREM1 | rs2234243 |
| CYP19A1 | rs3751592 | IL2RA | rs12244380 |  | PGRMC1 | rs2428757 | TREM1 | rs1817537 |
| CYP19A1 | rs3751591 | IL2RA | rs9663421 |  | PGRMC2 | rs11726595 | TREM1 | rs3804277 |
| CYP19A1 | rs2470150 | IL2RA | rs12722596 |  | PGRMC2 | rs2036687 | TREM1 | rs4711668 |
| CYP19A1 | rs1902584 | IL2RA | rs2386841 |  | PGRMC2 | rs4975220 | TREM1 | rs6910730 |
| CYP19A1 | rs1004983 | IL2RA | rs12722588 |  | PGRMC2 | rs4975180 | TREM1 | rs6940092 |
| CYP19A1 | rs1004982 | IL2RA | rs2076846 |  | PGRMC2 | rs3733260 | TREM1 | rs6939973 |
| CYP19A1 | rs1902585 | IL2RA | rs7093069 |  | PLA2G4A | rs3820185 | TREM1 | rs3827632 |
| CYP19A1 | rs10163138 | IL2RA | rs942200 |  | PLA2G4A | rs4651330 | TREM1 | rs3789204 |
| CYP19A1 | rs7174997 | IL2RA | rs2031229 |  | PLA2G4A | rs2076075 | TREM1 | rs1385105 |
| CYP19A1 | rs8025191 | IL2RA | rs2228150 |  | PLA2G4A | rs6696406 | TSHR | rs8009058 |
| CYP19A1 | rs1961177 | IL2RA | rs12722563 |  | PLA2G4A | rs12404877 | TSHR | rs2268451 |
| CYP1A1 | rs4646421 | IL2RA | rs7910961 |  | PLA2G4A | rs12720497 | TSHR | rs12892567 |
| CYP1A1 | rs2470893 | IL2RA | rs6602392 |  | PLA2G4A | rs6685652 | TSHR | rs179247 |
| CYP2D6 | rs5758589 | IL2RA | rs7072398 |  | PLA2G4A | rs2223307 | TSHR | rs179259 |
| CYP2D6 | rs764481 | IL2RA | rs11256457 |  | PLA2G4A | rs17591814 | TSHR | rs179260 |
| DHFR | rs12517451 | IL2RA | rs6602398 |  | PLA2G4A | rs2223309 | TSHR | rs179261 |
| DHFR | rs1650723 | IL2RA | rs11598648 |  | PLA2G4A | rs1980444 | TSHR | rs7160338 |
| DHFR | rs1677693 | IL2RA | rs4749926 |  | PLA2G4A | rs2049963 | TSHR | rs2110696 |
| DHFR | rs10072026 | IL2RA | rs1107345 |  | PLA2G4A | rs10911946 | TSHR | rs1035145 |
| DHFR | rs1650697 | IL2RA | rs11256497 |  | PLA2G4A | rs12749354 | TSHR | rs3783950 |
| DHFR | rs380691 | IL2RA | rs791589 |  | PLA2G4A | rs7540602 | TSHR | rs3783948 |
| EDN2 | rs12069358 | IL2RA | rs791590 |  | PLA2G4A | rs6695515 | TSHR | rs7143071 |
| EDN2 | rs1407550 | IL2RA | rs7089855 |  | PLA2G4A | rs726706 | TSHR | rs17111361 |
| EDN2 | rs6690839 | IL2RA | rs2476491 |  | PLA2G4A | rs6656909 | TSHR | rs6574616 |
| EDN2 | rs734359 | IL2RA | rs706779 |  | PLA2G4A | rs1569479 | TSHR | rs724169 |
| EDN2 | rs11572340 | IL2RA | rs706778 |  | PLA2G4A | rs6683515 | TSHR | rs2300520 |
| EDN2 | rs3754287 | IL2RA | rs3134883 |  | PLA2G4A | rs12726519 | TSHR | rs4903964 |
| [EDN2](http://snpper.chip.org/bio/show-gene/561) | rs1077218 | IL2RA | rs12722486 |  | PLA2G4A | rs6683416 | TSHR | rs917986 |
| EDN2 | rs883304 | IL2RA | rs7072793 |  | PLA2G4A | rs11587539 | TSHR | rs17545310 |
| EDN2 | rs12718439 | IL2RA | rs7073236 |  | PLA2G4A | rs7555140 | TSHR | rs3783943 |
| EDN2 | rs4660541 | IL2RB | rs2743827 |  | PLA2G4A | rs932476 | TSHR | rs8012937 |
| EPHX1 | rs2854450 | IL2RB | rs228937 |  | PLA2G4A | rs10157410 | TSHR | rs10483973 |
| EPHX1 | rs3753658 | IL2RB | rs3218339 |  | PLA2G4A | rs7545121 | TSHR | rs2300521 |
| EPHX1 | rs1877724 | IL2RB | rs3218329 |  | PLA2G4A | rs4402086 | TSHR | rs12881268 |
| EPHX1 | rs2671272 | IL2RB | rs84458 |  | PLA2G4A | rs7526089 | TSHR | rs2268466 |
| EPHX1 | rs1051740 | IL2RB | rs84460 |  | PLA2G4A | rs761517 | TSHR | rs4903967 |
| EPHX1 | rs2260863 | IL2RB | rs228945 |  | PLAT/tPA | rs4581040 | TSHR | rs7161100 |
| EPHX1 | rs2740168 | IL2RB | rs228947 |  | PLAT/tPA | rs4471024 | TSHR | rs2300525 |
| EPHX1 | rs2740170 | IL2RB | rs2072861 |  | PLAT/tPA | rs2020922 | TSHR | rs2110697 |
| EPHX1 | rs2234922 | IL2RB | rs3218315 |  | PLAT/tPA | rs879293 | TSHR | rs1005292 |
| EPHX1 | rs1051741 | IL2RB | rs3218312 |  | PLAT/tPA | rs2299609 | TSHR | rs722540 |
| EPHX1 | rs3753663 | IL2RB | rs228953 |  | PLAT/tPA | rs8178703 | TSHR | rs17111394 |
| EPHX1 | rs360063 | IL2RB | rs3218297 |  | PLAT/tPA | rs7837156 | TSHR | rs2268474 |
| EPHX2 | rs7837347 | IL2RB | rs228954 |  | PLAT/tPA | rs2020919 | TSHR | rs2300528 |
| EPHX2 | rs4149239 | IL2RB | rs3218295 |  | POMC | rs6713532 | TSHR | rs2284735 |
| EPHX2 | rs2741334 | IL2RB | rs3218292 |  | PON1 | rs854547 | TSHR | rs2075173 |
| EPHX2 | rs17057288 | IL2RB | rs228957 |  | PON1 | rs8491 | TSHR | rs17111431 |
| EPHX2 | rs7816586 | IL2RB | rs2281094 |  | PON1 | rs854548 | TSHR | rs10129380 |
| EPHX2 | rs891401 | IL2RB | rs228968 |  | PON1 | rs3735590 | TSHR | rs17111481 |
| EPHX2 | rs721619 | IL2RB | rs1362904 |  | PON1 | rs854551 | TSHR | rs7150670 |
| EPHX2 | rs10503812 | IL2RB | rs1003694 |  | PON1 | rs854552 | TSHR | rs2268475 |
| EPHX2 | rs17057312 | IL2RB | rs2235330 |  | PON1 | rs854555 | TSHR | rs11159491 |
| EPHX2 | rs4149252 | IL2RB | rs3218264 |  | PON1 | rs3917550 | TSHR | rs2024426 |
| EPHX2 | rs4149253 | IL2RB | rs228975 |  | PON1 | rs2269829 | TSHR | rs11845715 |
| EPHX2 | rs13269963 | IL2RB | rs3218258 |  | PON1 | rs3917542 | TSHR | rs12885526 |
| EPHX2 | rs1042064 | IL4 | rs2243248 |  | PON1 | rs662 | TSHR | rs8017455 |
| EPHX2 | rs4149259 | IL4 | rs2070874 |  | PON1 | rs3917538 | TSHR | rs917984 |
| EPHX2 | rs4149260 | IL4 | rs2227284 |  | PON1 | rs854560 | TSHR | rs7158881 |
| EPHX2 | rs7341557 | IL4 | rs2243261 |  | PON1 | rs854561 | TSHR | rs6574629 |
| EPHX2 | rs2640726 | IL4 | rs2243263 |  | PON1 | rs2272365 | TSHR | rs2268476 |
| FII | rs2070852 | IL4 | rs2243268 |  | PON1 | rs3917490 | TSHR | rs3783938 |
| FII | rs3136485 | IL4 | rs2243274 |  | PON1 | rs2049649 | TSHR | rs17111530 |
| FV | rs2187952 | IL4 | rs2243290 |  | PON1 | rs2299260 | TSHR | rs930099 |
| FV | rs2420369 | IL4R | rs2057768 |  | PON1 | rs2299261 | TSHR | rs7157900 |
| FV | rs2213865 | IL4R | rs6498012 |  | PON1 | rs854568 | TSHR | rs1957547 |
| FV | rs3766103 | IL4R | rs4787948 |  | PON1 | rs2299262 | TSHR | rs2300540 |
| FV | rs12131397 | IL4R | rs3024530 |  | PON1 | rs854569 | TSHR | rs1991517 |
| FV | rs9332624 | IL4R | rs3024537 |  | PON1 | rs2237583 | TSHR | rs2268477 |
| FV | rs9332618 | IL4R | rs3024547 |  | PON1 | rs757158 | TSHR | rs7144481 |
| FV | rs6427197 | IL4R | rs3024548 |  | PON1 | rs17166818 | TSHR | rs17630128 |
| FV | rs4656687 | IL4R | rs3024560 |  | PON2 | rs11977702 | TSHR | rs2288493 |
| FV | rs1557572 | IL4R | rs2239349 |  | PON2 | rs9641164 | TSHR | rs12883801 |
| FV | rs6020 | IL4R | rs2239347 |  | PON2 | rs987539 | UGT1A1 | rs3755319 |
| FV | rs6035 | IL4R | rs3024585 |  | PON2 | rs2286232 | UGT1A1 | rs6742078 |
| FV | rs12120605 | IL4R | rs3024623 |  | PON2 | rs2299266 | UGT1A1 | rs4148324 |
| FV | rs6427198 | IL4R | rs4787423 |  | PON2 | rs2237585 | UGT1A1 | rs12479045 |
| FV | rs1894697 | IL4R | rs3024648 |  | PON2 | rs11976060 | UGT1A1 | rs4663971 |
| FV | rs9332575 | IL4R | rs3024658 |  | PON2 | rs2286233 | UGT1A1 | rs929596 |
| FV | rs9332569 | IL4R | rs3024668 |  | PON2 | rs11981433 | UGT1A1 | rs2302538 |
| FV | rs6022 | IL4R | rs3024670 |  | PON2 | rs7802018 | UGT1A1 | rs4148328 |
| FV | rs7545236 | IL4R | rs3024675 |  | PON2 | rs2299267 | UGT1A1 | rs11888492 |
| FV | rs7534848 | IL4R | rs3024676 |  | PON2 | rs730365 | UGT1A1 | rs10929303 |
| FV | rs9287095 | IL4R | rs2234898 |  | PON2 | rs43037 | UGT1A1 | rs8330 |
| FV | rs2298908 | IL4R | rs1805013 |  | PON2 | rs6978425 | UGT1A1 | rs4148329 |
| FV | rs6019 | IL4R | rs1805015 |  | PTCRA | rs16896153 | UGT1A1 | rs6717546 |
| FV | rs12755775 | IL4R | rs1801275 |  | PTCRA | rs9471960 | UGT1A1 | rs1500482 |
| FV | rs10489185 | IL4R | rs1805016 |  | PTCRA | rs9471966 | UGT1A1 | rs6719561 |
| FV | rs3917854 | IL4R | rs8832 |  | PTCRA | rs6901007 | UGT1A1 | rs4663972 |
| FVII | rs555212 | IL4R | rs1029489 |  | PTCRA | rs2234185 | UGT1A1 | rs7586006 |
| FVII | rs1475931 | IL4R | rs4787956 |  | PTGER2 | rs1390376 | UGT1A1 | rs1500477 |
| FVII | rs488703 | IL5 | rs743562 |  | PTGER2 | rs1254600 | VEGF | rs699947 |
| FVII | rs3211719 | IL5 | rs739719 |  | PTGER2 | rs1254593 | VEGF | rs833068 |
| FAS | rs983751 | IL5 | rs739718 |  | PTGER2 | rs708498 | VEGF | rs833069 |
| FAS | rs4934434 | IL5 | rs2069812 |  | PTGER2 | rs12147805 | VEGF | rs3025010 |
| FAS | rs3758483 | IL5 | rs2069807 |  | PTGER2 | rs708505 | VEGF | rs3025033 |
| FAS | rs4064 | IL6 | rs1880243 |  | PTGER2 | rs708506 | VEGF | rs3025035 |
| FAS | rs6586165 | IL6 | rs12700386 |  | PTGER3 | rs959 | VEGF | rs998584 |
| FAS | rs1571011 | IL6 | rs1800797 |  | PTGER3 | rs1327460 | VEGF | rs6900017 |
| FAS | rs9658727 | IL6 | rs1800795 |  | PTGER3 | rs6656853 |  |  |
| FAS | rs9658742 | IL6 | rs2069840 |  | PTGER3 | rs7530345 |  |  |
