# Supplementary material for: Preterm Birth in Caucasians Is Associated with Coagulation and Inflammation Pathway Gene Variants
Source: PLoS One. 2008 Sep 26;3(9):e3283. doi: 10.1371/journal.pone.0003283 (PMC2553267; doi:10.1371/journal.pone.0003283)
Supplement: Table S2 — (0.11 MB DOC) [file pone.0003283.s002.doc]

Supplemental Table S2. Maternal single locus association results

| Gene(s) | rs# | Case v Control P-Value | |
| --- | --- | --- | --- |
|  |  | Allele | Genotype |
| AP3M2 | rs4581040 | 0.04 | 0.13 |
| AP3M2 | rs4471024 | 0.05 | 0.03 |
| CCL2 | rs1024610 | 0.68 | 0.01 |
| COL1A1 | rs1061237 | 0.09 | 0.05 |
| COL1A2 | rs420257 | 0.03 | 0.13 |
| COL1A2 | rs389328 | 0.03 | 0.07 |
| COL1A2 | rs42524 | 0.04 | 0.09 |
| COL1A2 | rs2521205 | 0.02 | 0.07 |
| COL1A2 | rs42528 | 0.04 | 0.07 |
| COL1A2 | rs7804898 | 0.05 | 0.10 |
| COL3A1 | rs3134646 | 0.04 | 0.01 |
| COL5A1 | rs12005720 | 0.04 | 0.10 |
| COL5A1 | rs4842167 | 0.01 | 0.03 |
| COL5A1 | rs3811161 | 0.02 | 0.08 |
| COL5A2 | rs10497699 | 0.28 | 0.04 |
| CRHBP | rs32897 | 0.004 | 0.02 |
| CRHBP | rs10055255 | 0.00059 | 0.01 |
| CRHBP | rs1875999 | 0.003 | 0.02 |
| CRHBP | rs10514082 | 0.04 | 0.14 |
| CTLA4 | rs16840252 | 0.52 | 0.01 |
| CTLA4 | rs231777 | 0.54 | 0.02 |
| CYP19A1 | rs17703982 | 0.13 | 0.04 |
| CYP19A1 | rs2470152 | 0.06 | 0.01 |
| CYP2D6 | rs764481 | 0.01 | 0.02 |
| DHFR | rs1650723 | 0.02 | 0.05 |
| EPHX2 | rs4149239 | 0.05 | 0.15 |
| EPHX2 | rs891401 | 0.04 | 0.03 |
| EPHX2 | rs10503812 | 0.04 | 0.08 |
| EPHX2 | rs4149252 | 0.04 | 0.07 |
| EPHX2 | rs4149259 | 0.06 | 0.03 |
| EPHX2 | rs4149260 | 0.03 | 0.01 |
| FV | rs2187952 | 0.04 | 0.09 |
| FV | rs2420369 | 0.04 | 0.07 |
| FV | rs3766103 | 0.04 | 0.07 |
| FV | rs9332624 | 0.003 | 0.01 |
| FV | rs6020 | 0.02 | 0.02 |
| FVII | rs3211719 | 0.06 | 0.01 |
| HSD11B1 | rs4844488 | 0.06 | 0.05 |
| IL-10 | rs3024496 | 0.01 | 0.03 |
| IL-10 | rs1800872 | 0.01 | 0.01 |
| IL-10 | rs1800896 | 0.02 | 0.04 |
| IL-10RA | rs2512143 | 0.05 | 0.17 |
| IL-10RA | rs2229113 | 0.04 | 0.10 |
| IL-18 | rs5744280 | 0.03 | 0.05 |
| IL-1R1 | rs3917225 | 0.03 | 0.01 |
| IL-1R1 | rs949963 | 0.04 | 0.08 |
| IL-1R1 | rs3917273 | 0.04 | 0.09 |
| IL-1R1 | rs2110726 | 0.01 | 0.01 |
| IL-1R2 | rs4851522 | 0.05 | 0.07 |
| IL-1R2 | rs1108338 | 0.04 | 0.08 |
| IL-1R2 | rs3218979 | 0.05 | 0.19 |
| IL-1RAP | rs6800625 | 0.02 | 0.04 |
| IL-1RAP | rs11929157 | 0.04 | 0.07 |
| IL-1RAP | rs1024941 | 0.04 | 0.14 |
| IL-2RA | rs2031229 | 0.03 | 0.14 |
| IL-2RA | rs791589 | 0.05 | 0.20 |
| IL-4R | rs3024530 | 0.03 | 0.07 |
| IL-4R | rs3024548 | 0.04 | 0.07 |
| IL-4R | rs3024658 | 0.03 | 0.10 |
| IL-5 | rs739719 | 0.03 | 0.06 |
| IL-5 | rs739718 | 0.0041 | 0.01 |
| IL-6R | rs7549338 | 0.05 | 0.06 |
| IL-6R | rs4845625 | 0.04 | 0.11 |
| IL-6R | rs11265618 | 0.04 | 0.12 |
| IL-6R | rs2229238 | 0.05 | 0.13 |
| IL-6R | rs4072391 | 0.04 | 0.13 |
| KL | rs659117 | 0.05 | 0.23 |
| MMP1 | rs1939008 | 0.19 | 0.03 |
| MMP3 | rs520540 | 0.06 | 0.01 |
| MMP3 | rs645419 | 0.05 | 0.01 |
| MTHFD1 | rs1956545 | 0.17 | 0.02 |
| NAT1 | rs7017402 | 0.03 | 0.09 |
| NAT1 | rs9325827 | 0.01 | 0.05 |
| NAT1 | rs17126350 | 0.01 | 0.03 |
| NFKBIA | rs3138056 | 0.70 | 0.04 |
| NFKBIB | rs11575002 | 0.02 | 0.08 |
| PGRMC1 | rs2499043 | 0.03 | 0.07 |
| PLA2G4A | rs11587539 | 0.81 | 0.05 |
| PLA2G4A | rs7545121 | 0.54 | 0.05 |
| PLAT/tPA | rs2020922 | 0.03 | 0.10 |
| PLAT/tPA | rs879293 | 0.0023 | 2.00x10^-6^ |
| PLAT/tPA | rs2299609 | 0.06 | 0.01 |
| PLAT/tPA | rs7837156 | 0.09 | 0.03 |
| PON2 | rs730365 | 0.01 | 0.02 |
| PTGER3 | rs959 | 0.86 | 0.05 |
| PTGER3 | rs602383 | 0.12 | 0.01 |
| PTGER3 | rs578096 | 0.03 | 0.12 |
| PTGER3 | rs6670616 | 0.04 | 0.06 |
| PTGER3 | rs2421735 | 0.03 | 0.04 |
| PTGER3 | rs977214 | 0.17 | 0.0041 |
| PTGER3 | rs6665776 | 0.24 | 0.01 |
| PTGER3 | rs594454 | 0.001 | 0.004 |
| PTGER3 | rs2300161 | 0.05 | 0.05 |
| PTGER3 | rs5697 | 0.04 | 0.06 |
| PTGER3 | rs2072947 | 0.02 | 0.03 |
| PTGER3 | rs2300167 | 0.01 | 0.05 |
| PTGER3 | rs5693 | 0.04 | 0.07 |
| PTGER3 | rs5680 | 0.01 | 0.03 |
| PTGER3 | rs11209736 | 0.04 | 0.11 |
| PTGER3 | rs8179390 | 0.03 | 0.10 |
| PTGER3 | rs2817864 | 0.03 | 0.08 |
| PTGS1 | rs10306188 | 0.34 | 0.04 |
| SCNN1A | rs3764874 | 0.003 | 0.01 |
| TEX12/IL18 | rs5744222 | 0.01 | 0.05 |
| TIMP3 | rs130290 | 0.01 | 0.02 |
| TIMP3 | rs130293 | 0.01 | 0.03 |
| TIMP3 | rs130300 | 0.04 | 0.08 |
| TIMP3 | rs130301 | 0.04 | 0.05 |
| TLR2 | rs1898830 | 0.02 | 0.05 |
| TLR7 | rs179007 | 0.01 | 0.04 |
| TLR7 | rs179006 | 0.03 | 0.08 |
| TNFRSF1A | rs4149578 | 0.03 | 0.05 |
| TNFRSF1B | rs976881 | 0.03 | 0.02 |
| TNFRSF1B | rs1201157 | 0.14 | 0.05 |
| TNFRSF1B | rs235214 | 0.83 | 0.03 |
| TSHR | rs11845715 | 0.02 | 0.10 |
| TSHR | rs1957547 | 0.03 | 0.05 |
| TSHR | rs17630128 | 0.03 | 0.02 |
| TSHR | rs2288493 | 0.05 | 0.03 |
| TSHR | rs12883801 | 0.01 | 0.01 |
| UGT1A1 | rs7586006 | 0.76 | 0.03 |
| VEGF | rs6900017 | 0.05 | 0.09 |
