# Supplementary material for: Preterm Birth in Caucasians Is Associated with Coagulation and Inflammation Pathway Gene Variants
Source: PLoS One. 2008 Sep 26;3(9):e3283. doi: 10.1371/journal.pone.0003283 (PMC2553267; doi:10.1371/journal.pone.0003283)
Supplement: Table S3 — (0.10 MB DOC) [file pone.0003283.s003.doc]

Supplemental Table S3. Fetal single locus association results

| Gene(s) | rs# | Cases v Control P-Value | |
| --- | --- | --- | --- |
|  |  | Allele | Genotype |
| CBS | rs6586282 | 0.05 | 0.02 |
| CBS | rs6586283 | 0.06 | 0.04 |
| CBS | rs12329764 | 0.16 | 0.002 |
| CBS | rs2851391 | 0.05 | 0.03 |
| CBS | rs11701048 | 0.45 | 0.01 |
| COL1A2 | rs1800222 | 0.21 | 0.05 |
| COL1A2 | rs4266 | 0.12 | 0.05 |
| COL3A1 | rs1516454 | 0.35 | 0.01 |
| COL3A1 | rs3134656 | 0.01 | 0.02 |
| COL3A1 | rs2271682 | 0.10 | 0.05 |
| COL3A1 | rs2203602 | 0.03 | 0.12 |
| COL3A1 | rs3134646 | 0.01 | 0.05 |
| COL5A2 | rs13024858 | 0.05 | 0.07 |
| COL5A2 | rs1515864 | 0.04 | 0.04 |
| COL5A2 | rs9288163 | 0.03 | 0.03 |
| CRHBP | rs32897 | 0.28 | 0.05 |
| CRHBP | rs1875999 | 0.02 | 0.04 |
| CRHR2 | rs12701020 | 0.01 | 0.05 |
| CYP19A1 | rs2899473 | 0.03 | 0.07 |
| CYP19A1 | rs3784308 | 0.03 | 0.03 |
| CYP19A1 | rs17703982 | 0.03 | 0.05 |
| CYP19A1 | rs17647719 | 0.02 | 0.03 |
| CYP19A1 | rs1902586 | 0.02 | 0.02 |
| CYP19A1 | rs3764221 | 0.04 | 0.08 |
| CYP19A1 | rs2470152 | 0.03 | 0.12 |
| CYP19A1 | rs16964258 | 0.04 | 0.05 |
| CYP2D6 | rs764481 | 0.01 | 0.03 |
| EDN2 | rs12069358 | 0.87 | 0.03 |
| EDN2 | rs4660541 | 0.24 | 0.01 |
| EPHX2 | rs891401 | 0.03 | 0.08 |
| EPHX2 | rs10503812 | 0.03 | 0.12 |
| EPHX2 | rs4149252 | 0.04 | 0.15 |
| EPHX2 | rs4149259 | 0.02 | 0.05 |
| EPHX2 | rs4149260 | 0.02 | 0.07 |
| GSTP1 | rs947895 | 0.05 | 0.10 |
| HSPA14 | rs10906772 | 0.01 | 0.04 |
| HSPA6 | rs12129787 | 0.12 | 0.03 |
| IGF1 | rs1520220 | 0.43 | 0.05 |
| IL-10RA | rs4936414 | 0.01 | 0.06 |
| IL-10RA | rs2512143 | 0.01 | 0.03 |
| IL-10RA | rs2229113 | 0.02 | 0.04 |
| IL-10RA | rs9610 | 0.01 | 0.02 |
| IL-10RA | rs2508445 | 0.03 | 0.06 |
| IL-10RA | rs947889 | 0.02 | 0.06 |
| IL-10RA | rs4938467 | 0.04 | 0.13 |
| IL-10RA | rs11216666 | 0.01 | 0.01 |
| IL-10RA | rs17121510 | 0.01 | 3.34x10^-4^ |
| IL-1A | rs17561 | 0.01 | 0.04 |
| IL-1A | rs1878321 | 0.01 | 0.05 |
| IL-1B | rs1143630 | 0.04 | 0.03 |
| IL-1RAP | rs2059020 | 0.05 | 0.09 |
| IL-1RAP | rs2361832 | 0.01 | 0.03 |
| IL-1RAP | rs9883249 | 0.08 | 0.01 |
| IL-1RAP | rs9845825 | 0.02 | 0.01 |
| IL-1RAP | rs2241343 | 0.03 | 0.04 |
| IL-1RAP | rs3773977 | 0.21 | 0.04 |
| IL-1RN | rs315920 | 0.24 | 0.04 |
| IL-2RA | rs12722596 | 0.03 | 0.09 |
| IL-2RB | rs3218295 | 0.04 | 0.07 |
| IL-2RB | rs3218292 | 0.07 | 0.03 |
| IL-4 | rs2243248 | 0.04 | 0.09 |
| IL-4R | rs2239349 | 0.04 | 0.03 |
| IL-5 | rs739719 | 0.12 | 0.05 |
| KL | rs9536239 | 0.02 | 0.05 |
| KL | rs9527025 | 0.004 | 0.02 |
| KL | rs522796 | 0.003 | 0.002 |
| KL | rs582524 | 0.04 | 0.06 |
| MMP1 | rs1155764 | 0.01 | 0.02 |
| MMP3 | rs520540 | 0.05 | 0.10 |
| MMP3 | rs645419 | 0.03 | 0.09 |
| MMP8 | rs17099443 | 0.94 | 0.04 |
| MMP8 | rs6590985 | 0.73 | 0.01 |
| NFKBIA | rs3138045 | 1.00 | 0.02 |
| NFKBIB | rs11083487 | 0.05 | 0.14 |
| NOS3 | rs3918227 | 0.05 | 0.17 |
| NR3C1 | rs9324918 | 0.03 | 0.08 |
| NR3C1 | rs7701443 | 0.05 | 0.17 |
| PAFAH1B1 | rs4790355 | 0.02 | 0.02 |
| PAFAH1B1 | rs4790356 | 0.02 | 0.02 |
| PAFAH1B1 | rs2317297 | 0.02 | 0.02 |
| PAFAH1B1 | rs4790353 | 0.02 | 0.02 |
| PGR | rs471767 | 0.39 | 0.03 |
| PGR | rs492457 | 0.17 | 0.03 |
| PGR | rs538915 | 0.03 | 0.05 |
| PGR | rs555572 | 0.02 | 0.05 |
| PGR | rs11224589 | 0.02 | 0.09 |
| PGR | rs619487 | 0.01 | 0.04 |
| PLAT/tPA | rs879293 | 0.04 | 0.11 |
| PON1 | rs3917542 | 0.04 | 0.11 |
| PON1 | rs2272365 | 0.05 | 0.05 |
| PON2 | rs2286233 | 0.01 | 0.04 |
| PTGER3 | rs602383 | 0.04 | 0.11 |
| PTGS2 | rs689462 | 0.02 | 0.02 |
| SLC23A1 | rs6596471 | 0.04 | 0.10 |
| SLC35B2 | rs520639 | 0.02 | 0.04 |
| TIMP3 | rs242076 | 0.80 | 0.05 |
| TREM1 | rs4711668 | 0.02 | 0.03 |
| TREM1 | rs6910730 | 0.003 | 0.003 |
| TREM1 | rs3827632 | 0.02 | 0.02 |
| TREM1 | rs3789204 | 0.57 | 0.03 |
| TREM1 | rs1385105 | 0.04 | 0.04 |
| TSHR | rs179247 | 0.03 | 0.03 |
| UGT1A1 | rs11888492 | 0.05 | 0.11 |
