# Supplementary figures and images for: Preterm Birth in Caucasians Is Associated with Coagulation and Inflammation Pathway Gene Variants
Source: PLoS One. 2008 Sep 26;3(9):e3283. doi: 10.1371/journal.pone.0003283 (PMC2553267; doi:10.1371/journal.pone.0003283)

Figure S1. Maternal CRHBP Cases


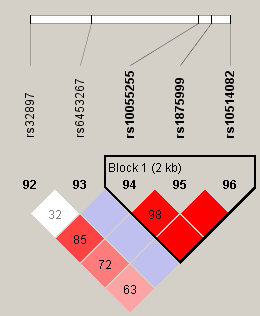

Supplement: Figure S1 — Maternal CRHBP Cases (0.03 MB DOC) [file pone.0003283.s004.doc]

Figure S2. Maternal CRHBP Controls


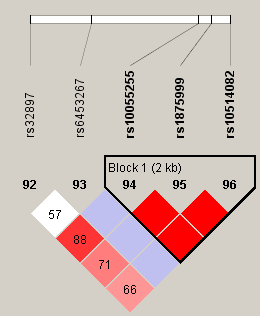

Supplement: Figure S2 — Maternal CRHBP Controls (0.03 MB DOC) [file pone.0003283.s005.doc]

Figure S3. Maternal IL-5 Cases


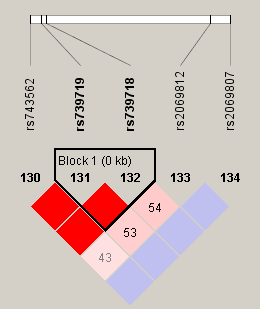

Supplement: Figure S3 — Maternal IL-5 Cases (0.03 MB DOC) [file pone.0003283.s006.doc]

Figure S4. Maternal IL-5 Controls


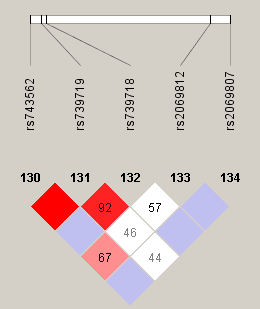

Supplement: Figure S4 — Maternal IL-5 Controls (0.03 MB DOC) [file pone.0003283.s007.doc]

Figure S5. Maternal tPA Cases


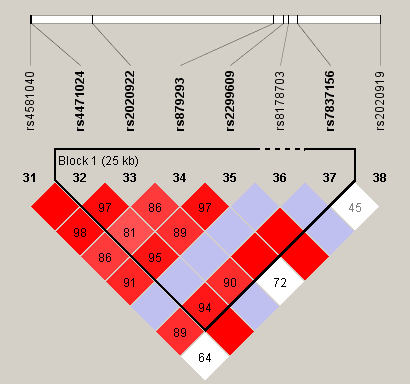

Supplement: Figure S5 — Maternal tPA Cases (0.03 MB DOC) [file pone.0003283.s008.doc]

Figure S6. Maternal tPA controls


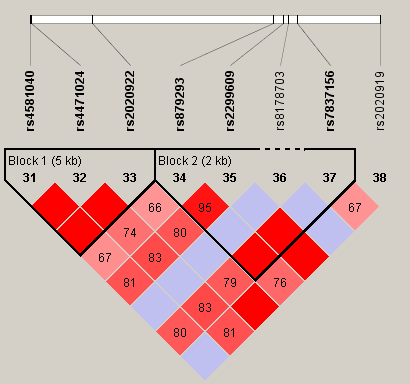

Supplement: Figure S6 — Maternal tPA controls (0.03 MB DOC) [file pone.0003283.s009.doc]

Figure S7. Maternal FV Cases


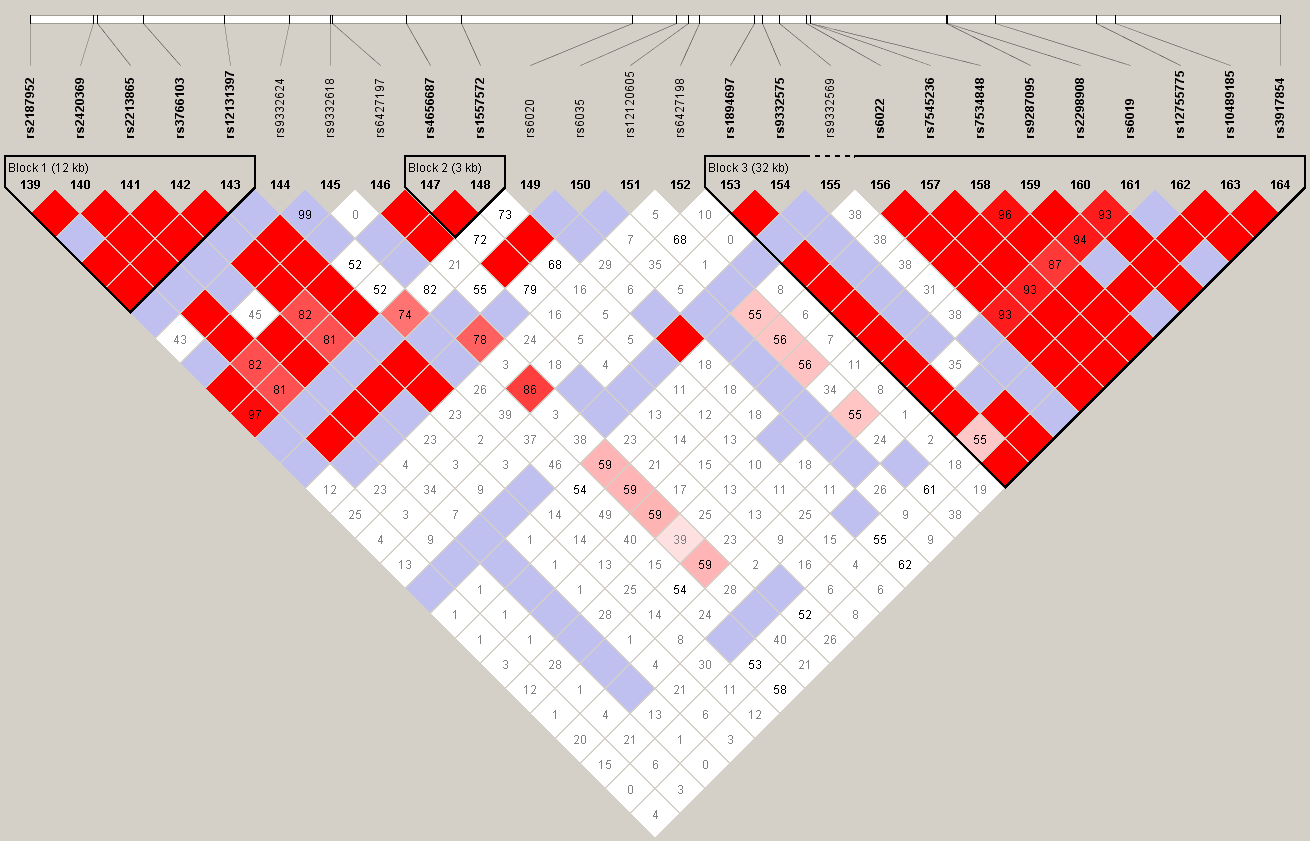

Supplement: Figure S7 — Maternal FV Cases (0.08 MB DOC) [file pone.0003283.s010.doc]

Figure S8. Maternal FV Controls


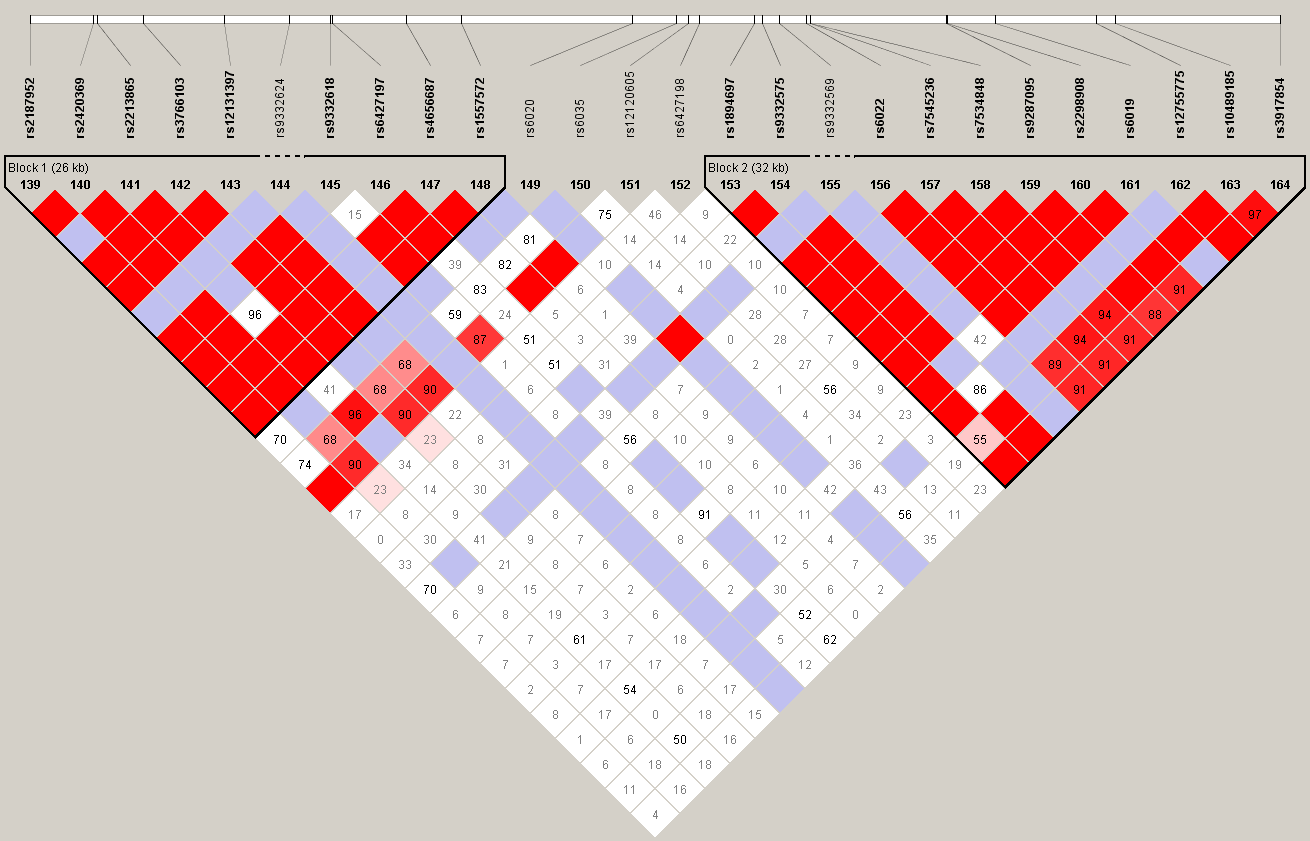

Supplement: Figure S8 — Maternal FV Controls (0.08 MB DOC) [file pone.0003283.s011.doc]

Figure S9. Maternal PTGER3 Cases


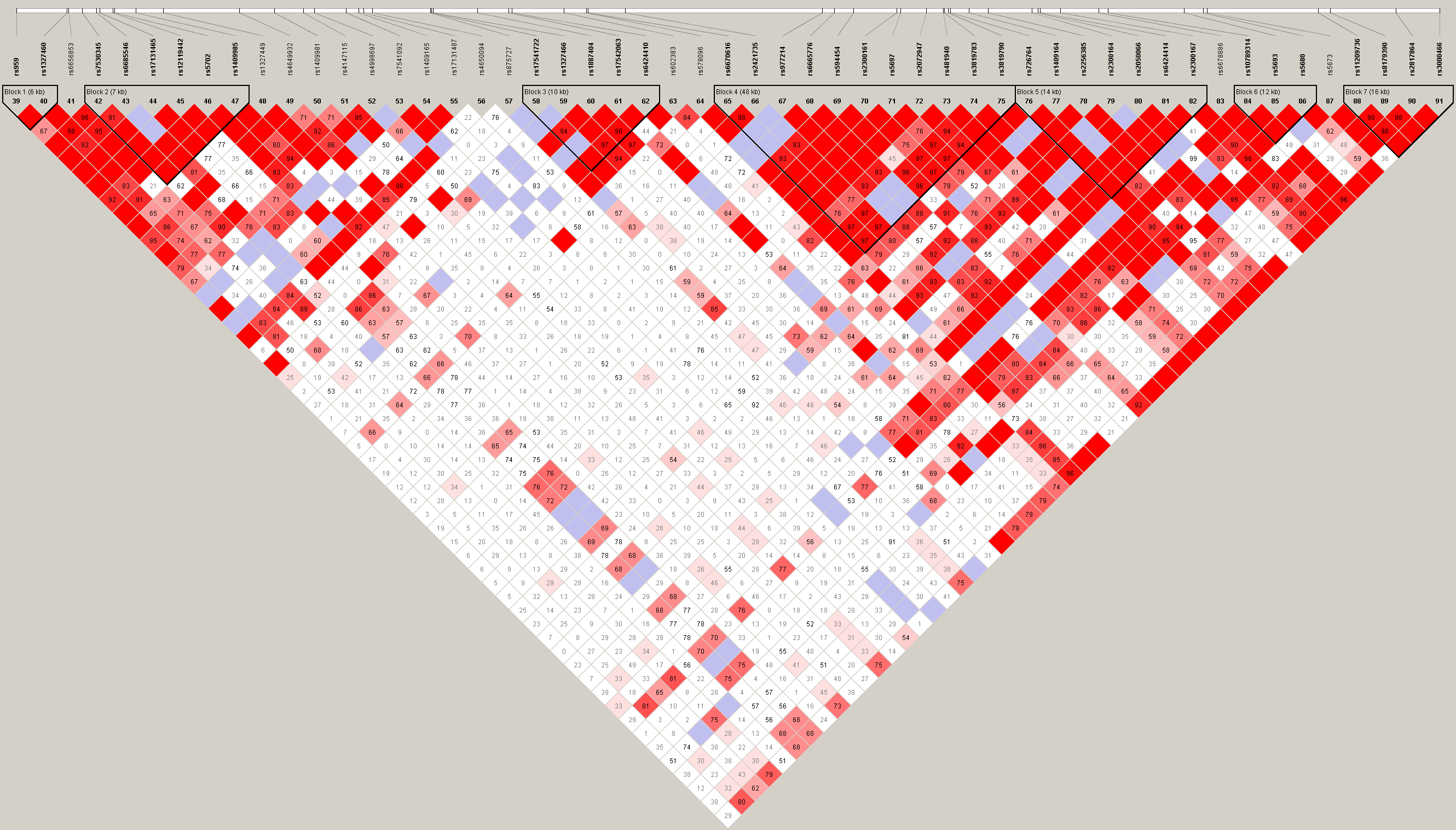

Supplement: Figure S9 — Maternal PTGER3 Cases (0.24 MB DOC) [file pone.0003283.s012.doc]

Figure S10. Maternal PTGER3 Controls


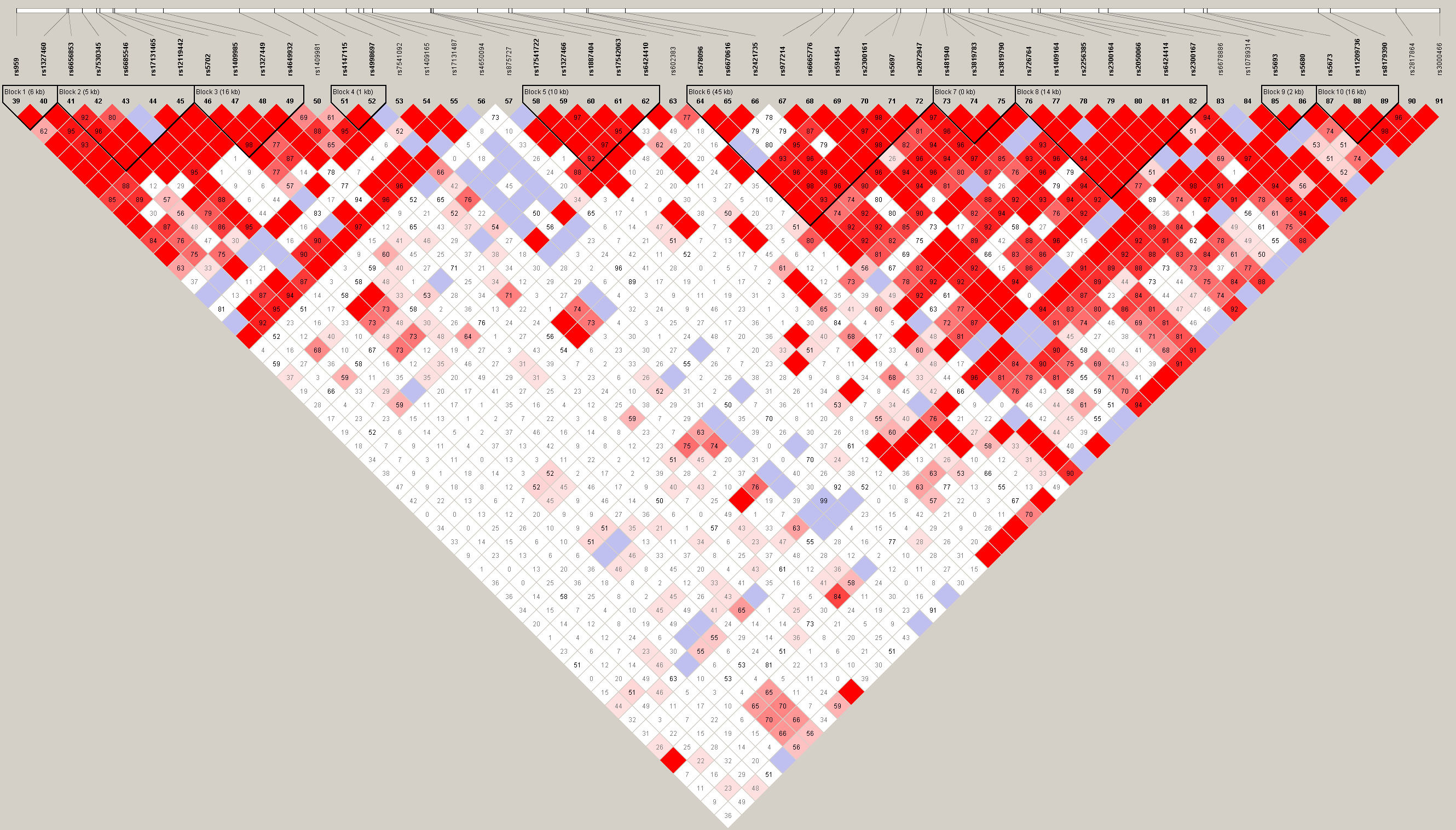

Supplement: Figure S10 — Maternal PTGER3 Controls (0.24 MB DOC) [file pone.0003283.s013.doc]

Figure S11. Fetal CBS Cases


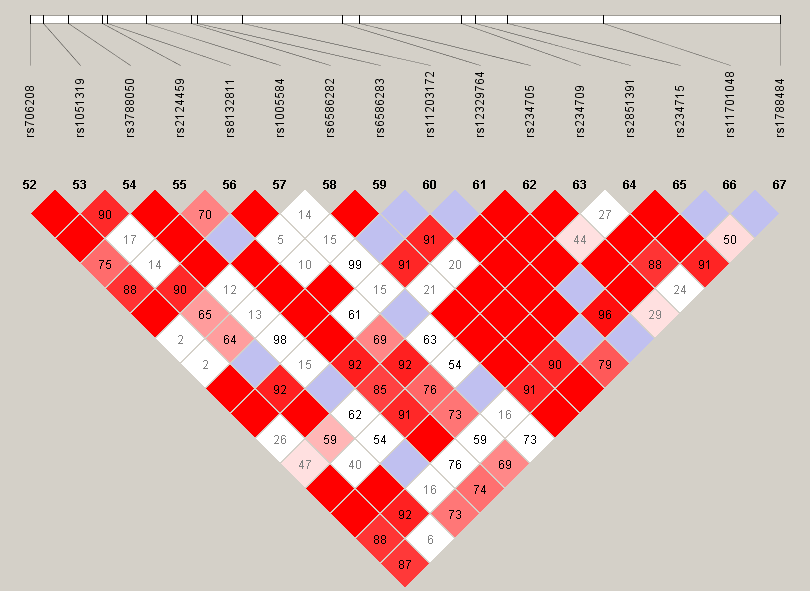

Supplement: Figure S11 — Fetal CBS Cases (0.05 MB DOC) [file pone.0003283.s014.doc]

Figure S12. Fetal CBS Controls


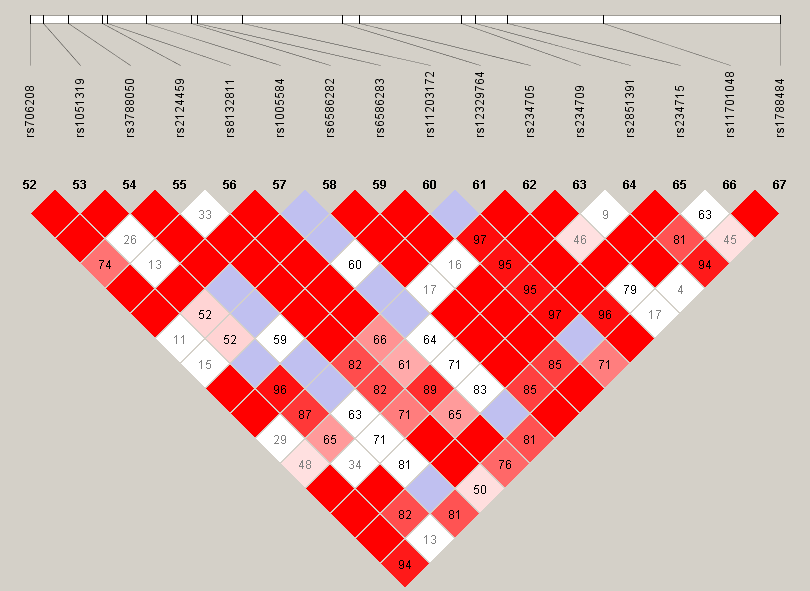

Supplement: Figure S12 — Fetal CBS Controls (0.05 MB DOC) [file pone.0003283.s015.doc]

Figure S13. Fetal IL-10RA Cases


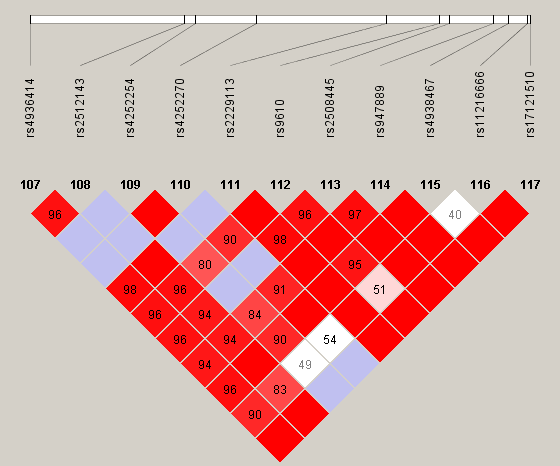

Supplement: Figure S13 — Fetal IL-10RA Cases (0.04 MB DOC) [file pone.0003283.s016.doc]

Figure S14. Fetal IL-10RA Controls


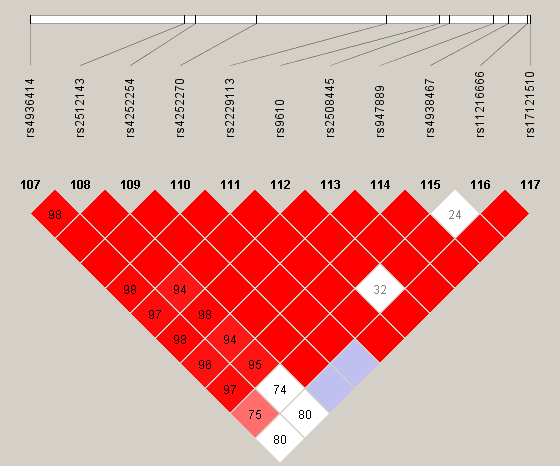

Supplement: Figure S14 — Fetal IL-10RA Controls (0.04 MB DOC) [file pone.0003283.s017.doc]

Figure S15. Fetal TREM1 Cases


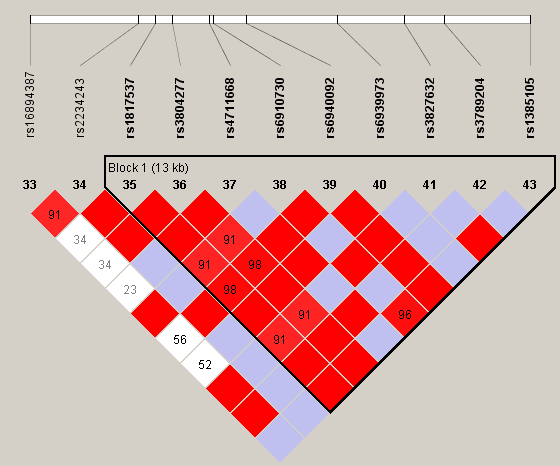

Supplement: Figure S15 — Fetal TREM1 Cases (0.04 MB DOC) [file pone.0003283.s018.doc]

Figure S16. Fetal TREM1 Controls


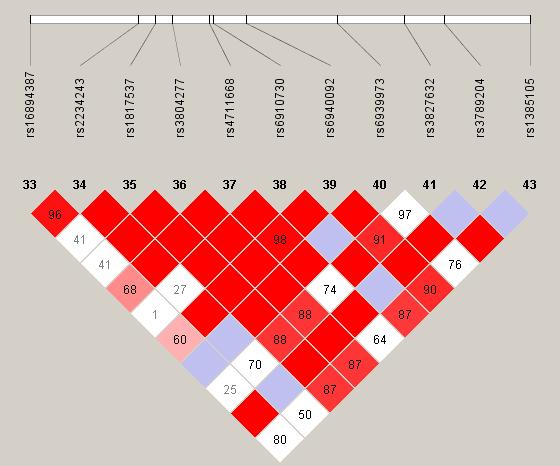

Supplement: Figure S16 — Fetal TREM1 Controls (0.04 MB DOC) [file pone.0003283.s019.doc]
